# Hyperfusogenic Mutations Destabilize the Postfusion Six-Helix Bundle of the Measles Virus Fusion Glycoprotein

**DOI:** 10.1021/acs.biochem.6c00182

**Published:** 2026-07-22

**Authors:** Nisansala Vithanage, Victor K. Outlaw

**Affiliations:** Department of Chemistry, 14716 University of Missouri, Columbia, Missouri 65211, United States

## Abstract

Fusion of the host membrane and viral envelope by class I viral fusion proteins is driven by the assembly of a postfusion six-helix bundle formed through antiparallel interactions between N-terminal (HR1) and C-terminal (HR2) heptad-repeat regions. Although mutations in these regions of the measles virus (MeV) fusion (F) glycoprotein are known to promote neuropathogenic and hyperfusogenic phenotypes, their effects on postfusion core stability have not been systematically examined. Here, we combine peptide biophysics and X-ray crystallography to interrogate how mutations within the HR2 domain, present in native neuropathogenic MeV isolates (*e.g*., L454W and N462K) and laboratory-generated hyperfusogenic variants (*e.g*., L454M and T461A), influence postfusion 6HB assembly. Circular dichroism (CD) spectroscopy reveals that, with few exceptions, these mutations decrease postfusion core stability, despite their association with enhanced fusion activity. We also report the first crystal structure of the wild-type MeV postfusion core as well as structures of six hyperfusogenic variants, enabling high-resolution comparison of the molecular basis of destabilization. Structural analysis shows that these effects arise from localized perturbations to steric packing, hydrogen bonding networks, and helix-stabilizing interactions within HR2, while the overall 6HB architecture remains conserved. Together, these results indicate that hyperfusogenic mutations are not associated with stabilization of the postfusion state and are instead consistent with models in which hyperfusogenicity arises from a reduction in the energetic barrier to fusion, potentially through effects on prefusion stability or triggering efficiency. These findings establish key sequence-structure–stability relationships governing coiled-coil assembly and provide a framework for the design of HR1- and HR2-based fusion inhibitors.

## Graphical abstract

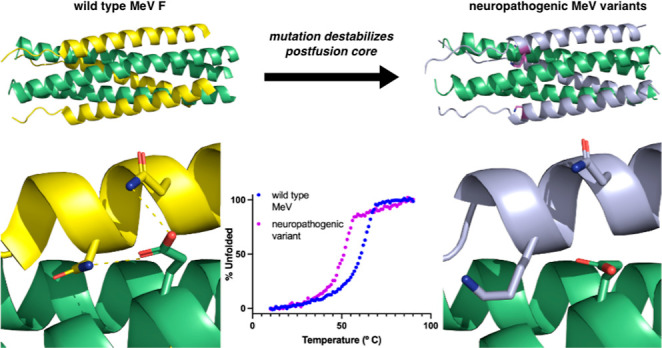

## Introduction

Membrane fusion mediated by class I viral fusion proteins is driven by a sequence of large-scale conformational changes that culminate in the formation of a highly stable postfusion assembly. Central to this process is the interaction between N-terminal (HR1) and C-terminal (HR2) heptad repeat domains, which associate to form an antiparallel trimer-of-hairpins architecture or six-helix bundle (6HB, Figure 1).^1–7^ This HR1-HR2-mediated coiled-coil assembly represents the thermodynamic end point of fusion and is conserved across a broad range of enveloped viruses.^8,9^ As such, HR1-HR2 interactions provide a well-defined and chemically tractable system for probing how sequence variation modulates coiled-coil assembly, stability, and function.

**Figure 1:**
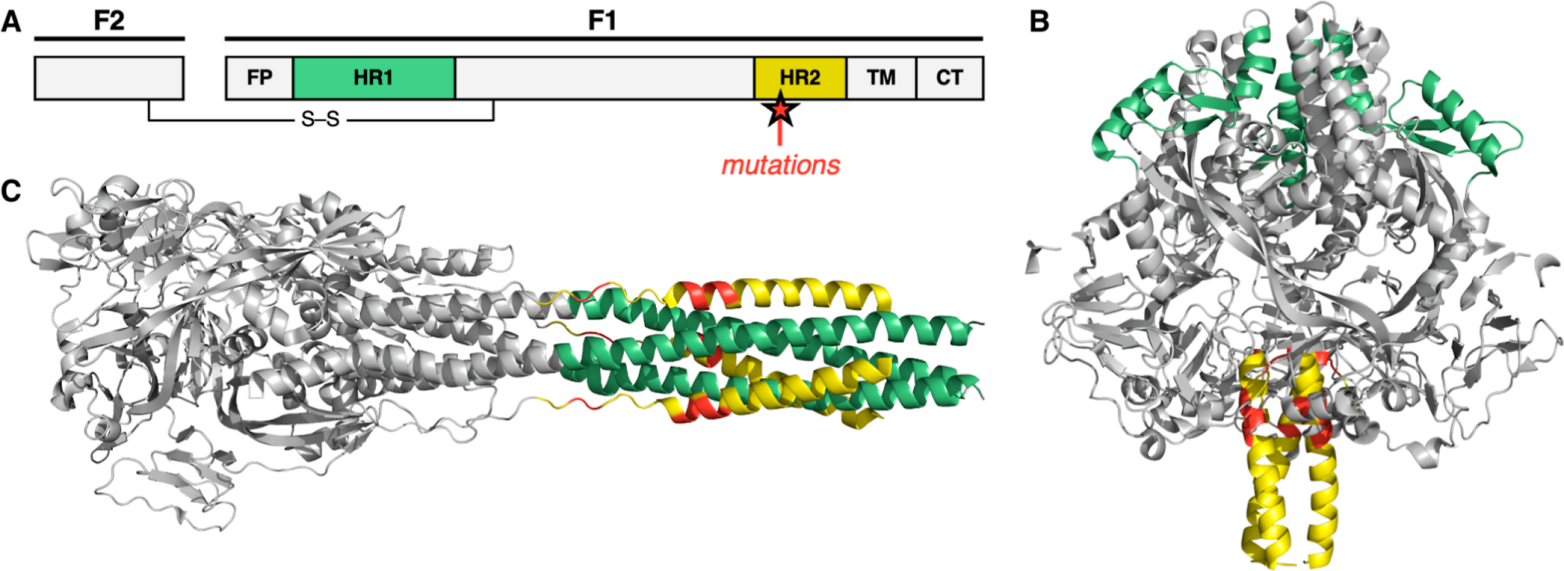
Domain architecture and structure of MeV F. (A) Schematic diagram of MeV depicting the F1 and F2 subunits and the fusion peptide (FP), N-terminal heptad repeat (HR1), C-terminal heptad repeat (HR2), transmembrane domain (TM), and cytoplasmic tail (CT) domains. (B) X-ray structure of MeV F prefusion conformation (PDB: 5YXW). (C) Cryo-EM structure of MeV ectodomain postfusion conformation (PDB: 8UTF. Select regions are colored to highlight the HR1 (green), HR2 (yellow), and neuropathogenic/hyperfusogenic HR2 mutations (red).

The measles virus (MeV) fusion (F) glycoprotein is a prototypical class I fusion protein and widely studied model for paramyxovirus membrane fusion.^10–12^ Initially synthesized as an inactive single-chain precursor (F_0_), proteolytic cleavage generates a metastable prefusion F conformation. Engagement of cell surface receptors, such as CD150 (SLAM) or Nectin-4, by MeV hemagglutinin (H) leads to activation of F, which undergoes irreversible refolding and results in 6HB formation through HR1-HR2 assembly.^13–18^ Synthetic peptides derived from the HR2 region act as competitive inhibitors by binding to exposed HR1 segments and preventing native 6HB assembly, underscoring the critical role of these heptad repeat interactions in driving fusion and highlighting their relevance to peptide-based inhibitor design.^19–24^

Over the last two decades, mutations within the HR2 domain of MeV F have been identified that contribute to neuropathogenic and hyperfusogenic phenotypes.^25–37^ These substitutions, such as L454W in clinical isolates, as well as laboratory-derived mutations, such as T461A, are known to destabilize the prefusion conformation of F, thereby lowering the barrier for activation. The impact of these mutations on the stability of the postfusion 6HB assembly, however, remains unexplored.

From a peptide chemistry perspective, these HR2 mutations represent targeted perturbations to a coiled-coil assembly in which side chain sterics, charge, polarity, hydrogen-bond capacity, and α-helix propensity are expected to play critical roles in determining stability. In prior work, we demonstrated that mutations within the heptad repeat domains of the SARS-CoV-2 spike glycoprotein, such as Omicron variant Q954H, enhance postfusion core stability through localized changes in postfusion core interactions.^38^ These findings demonstrated that sequence variation within heptad repeat regions can modulate postfusion assembly energetics in a manner that directly influences membrane fusion efficiency. Because HR1-HR2 association contributes to the thermodynamic driving force for fusion, it is reasonable to hypothesize that hyperfusogenic mutations might similarly stabilize the postfusion assembly, thereby reinforcing fusion progression. However, direct experimental evidence of this hypothesis is limited, and the relationship between hyperfusogenic MeV mutations and postfusion thermodynamic stability remains poorly defined.

Despite the central importance of HR1-HR2 interactions, a crystallographic structure of the postfusion core of wild-type MeV F has not previously been reported. Existing structural information has relied on homologous paramyxovirus systems,^39–42^ limiting direct evaluation of how sequence variation influences MeV postfusion assembly. Here, we report the first crystal structure of the wild-type MeV postfusion core, as well as structures of a series of neuropathogenic and hyperfusogenic variants. By combining circular dichroism (CD) spectroscopy with high-resolution X-ray crystallography, we systematically assess how HR2 mutations alter postfusion 6HB stability and structure. Our results reveal that, contrary to expectation, hyperfusogenic mutations consistently *decrease* postfusion core stability while preserving the overall 6HB architecture. These findings decouple hyperfusogenicity from postfusion thermodynamic stabilization and establish general principles governing peptide-mediated coiled-coil assembly with direct implications for the rational design of HR1 -and HR2-derived fusion inhibitors.

## Experimental Procedures

### Peptide Synthesis

Peptides were synthesized using a Rink amide resin by standard Fmoc solid-phase peptide synthesis protocols. All peptides were acetylated on the N-termini and contained C-terminal amides. Synthetic procedures and characterization data are provided in the Supporting Information.

### Circular Dichroism Spectroscopy

All CD experiments were performed on a JASCO J-1500 CD spectrophotometer using 1 mm high-performance quartz glass cuvettes (Hellma) with a spectral range of 200–2500 nm and a chamber volume of 400 μL.

For wavelength scan experiments, CD samples were prepared using a 1:1 mixtures of MeV HR1-wt (0.5–1 μg/μL) and HR2 peptide variants (0.5–1 μg/μL) at 25 μM concentrations in PBS (pH 7.0) with 2.5% hexafluoro-2-propanol (HFIP). Wavelength scans were collected at 25 °C from 250 to 200 nm with a 1 nm bandwidth. Experiments were performed in triplicate.

For variable-temperature experiments, CD samples were prepared using 1:1 mixture of MeV HR1-wt (0.5–1 μg/μL) and HR2 peptide variants (0.5–1 μg/μL) at 25 μM concentrations in PBS (pH 7.0) with 2.5% hexafluoro-2-propanol (HFIP). Variable-temperature CD spectra for each coassembly were performed by monitoring ellipticity at 222 nm as a function of temperature from 10–90 °C in 2 °C increments with a 1.00 °C/min rate, 20 s equilibration time, and a 0.15 °C tolerance. All experiments were performed in triplicate. Data were fit to a two-state folding model using GraphPad Prism 10, and apparent melting temperature (*T*_m,app_) values, defined as the midpoint of each transition, were determined. Experiments were performed in triplicate.

### X-ray Crystallography

A cocrystallization solution was prepared containing a 1:1 mixture of MeV HR1-wt and HR2 peptide variants in water at a total concentration of 5–12 mg/mL. Crystals were grown by hanging drop vapor diffusion using a crystallization condition optimized from the Morpheus crystallization screen (Molecular Dimensions). A 2 μL drop comprising a 1:1 mixture of cocrystallization solution and the crystallization condition was placed on a glass cover slide that was then inverted to seal a well containing 150 μL of the crystallization condition. Crystallization conditions for each structure are provided in Table S2.

The crystals were looped and vitrified in liquid nitrogen. Diffraction data were collected at the National Synchrotron Light Source II (NSLS-II) at Brookhaven National Laboratory (BNL) on the AMX beamline (17-ID-1). Data were indexed and integrated using the program *XDS*.^57^ Data were scaled and merged using the program *AutoProc*.^58^ The molecular replacement solutions were found with the program *Phaser* using search models provided in Table S3 as search models. Model refinement was carried out using the program *phenix.refine* ^59^ in combination with manual real-space model building and refinement in the program *Coot*.^60^

## Results and Discussion

### Design and Assembly of HR1-HR2 Peptide Coiled-Coils

To enable systematic evaluation of how individual side chain substitutions influence postfusion assembly, we employed a peptide-based model of the MeV fusion core that isolates HR1-HR2 interactions from cellular and prefusion triggering effects. Peptides corresponding to the HR1 and HR2 domains of MeV F were designed with lengths sufficient to support stable coiled-coil formation while minimizing excess hydrophobicity that could promote nonspecific aggregation (Figure 2). A 48-residue peptide spanning residues 142–189 of HR1 (HR1-wt) was selected based on sequence conservation across all MeV variants. A complementary 36-residue peptide spanning residues 452–487 of HR2 (HR2-wt) was synthesized along with a panel of variants bearing substitutions associated with neuropathogenic or hyperfusogenic phenotypes and targeted alanine controls. Peptide lengths were selected based on prior studies of related paramyxovirus systems (e.g., HPIV3 ^41,42^ ) to capture the full structural elements involved in fusion core formation, including both the helical and N-terminal extended regions of HR2.

**Figure 2:**
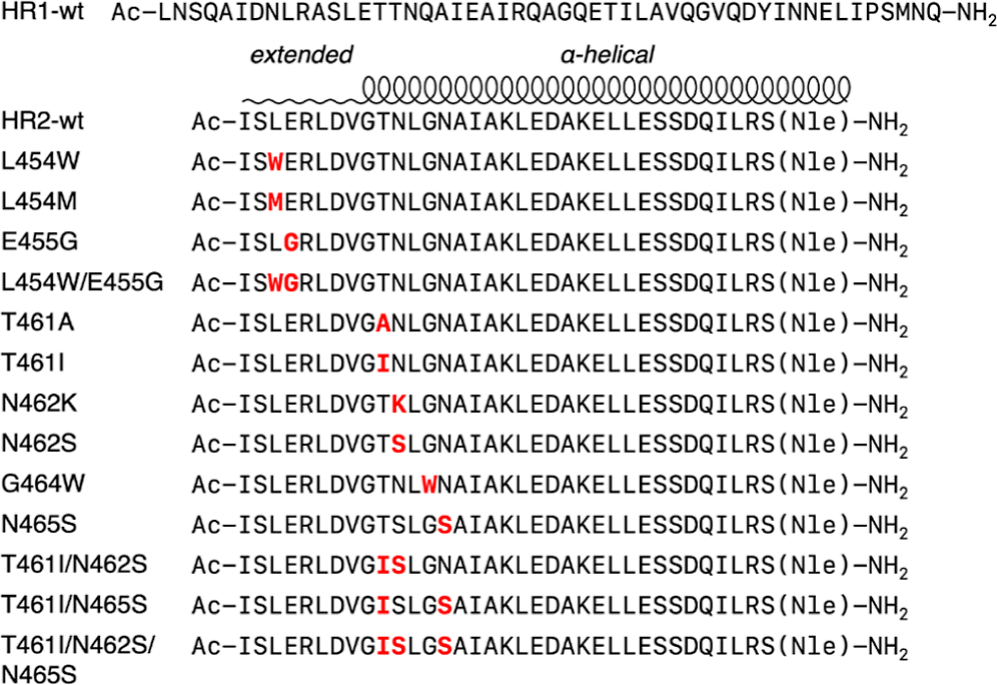
Sequences of synthetic peptides to probe the impact of MeV neuropathogenic and hyperfusogenic mutations on HR1-HR2 coiled-coil stability and structure.

Peptide sequences were engineered to permit a direct comparison of assembly energetics across variants. All peptides were N-terminally acetylated and C-terminally amidated to neutralize terminal charges and better mimic the native helical environment of the full-length protein. In the HR2 peptides, the C-terminal methionine residue was replaced with norleucine (Nle), an isosteric and oxidation-resistant analogue, to ensure chemical stability during synthesis and biophysical characterization without significantly altering hydrophobic character. These modifications allowed differences in thermal stability to be attributed primarily to side-chain substitutions within the HR2 region rather than to extrinsic chemical effects.

HR1-HR2 complexes were formed by equimolar mixing under aqueous conditions optimized to promote coiled-coil assembly while maintaining optical clarity for spectroscopic measurements. Because the HR1 peptide exhibits high intrinsic hydrophobicity, aggregation was mitigated by inclusion of a minimal fraction of hexafluoro-2-propanol (HFIP; 2.5% v/v) during biophysical analysis. While HFIP can influence peptide structure at higher concentrations, low percentages are commonly used in CD studies of hydrophobic peptides and are not expected to significantly perturb coiled-coil structure. Consistent with this, all HR1-HR2 assemblies examined here exhibited similar α-helical CD signatures and cooperative unfolding behavior under these conditions.^43–45^ This peptide-based system, therefore, provides a robust platform for directly assessing how targeted HR2 mutations modulate coiled-coil assembly and, therefore, postfusion core stability.

### 6HB Architecture Preserved across HR1-HR2 Variants

CD spectroscopy was used to evaluate secondary structure formation and thermal unfolding behavior of HR1-HR2 complexes assembled from wild-type and variant HR2 peptides. Representative CD wavelength scans and thermal denaturation profiles are shown in Figure 3, while melting temperatures for all variants are summarized in Table 1, and complete data sets are provided in the Supporting Information. All complexes exhibited characteristic α-helical CD signatures, with pronounced minima at 208 and 222 nm, consistent with formation of coiled-coil assemblies resembling the postfusion 6HB. The similarity of wavelength-dependent spectra across all variants indicates that the introduced substitutions do not disrupt the ability of HR1 and HR2 peptides to adopt helical conformations or to associate into the canonical 6HB architecture.

**Figure 3:**
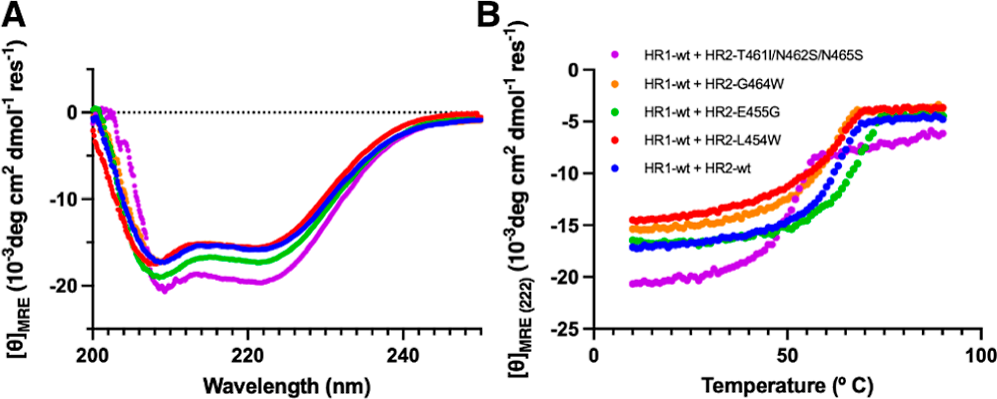
Representative circular dichroism studies of MeV wild-type and selected variant fusion core assemblies. (A) Wavelength scans of 1:1 mixtures of HR1-wt with HR2-wt (blue), HR2-L454W (red), HR2-E455G (green), HR2-G464W (orange), and HR2-T461I/N462S/N465S (purple) at 25 μM in PBS (pH 7.0) demonstrate conserved α-helical secondary structure. (B) Temperature-dependent denaturation of 1:1 mixtures of HR1-wt with HR2-wt (blue), HR2-L454W (red), HR2-E455G (green), HR2-G464W (orange), and HR2-T461I/N462S/N465S (purple) at 25 μM in PBS (pH 7.0)+2.5% HFIP. Complete data sets for all variants are provided in the Supporting Information.

**Table 1:** Apparent Melting Temperatures of MeV Fusion Core Assemblies

MeV variant^a^	*T*_m,app_ (°C)^b^	Δ*T*_m,app_ (°C)^c^
wild type	60.6(±0.3)	-
L454W	57.2(±0.5)	–3.4
L454M	57.6(±0.4)	–3.0
E455G	65.1(±0.2)	+4.5
L454W/E455G	61.5(±0.5)	+0.9
T461A	57.7(±0.4)	–2.9
T461I	60.1(±0.4)	–0.5
N462K	55.0(±0.2)	–5.6
N462S	55.3(±0.2)	–5.3
N465S	56.1(±0.2)	–4.5
T461I/N462S	54.9(±0.2)	–5.7
T461I/N465S	56.4(±0.3)	–4.2
T461I/N462S/N465S	50.3(±0.5)	–10.3
G464W	57.7(±0.3)	–2.9

a Coiled-coil assemblies were created by using 1:1 mixtures of MeV HR1-wt and MeV HR2 peptide variants. Variants listed indicate mutations within the MeV HR2 peptide.

b Variable-temperature circular dichroism (vt-CD) experiments were performed by monitoring ellipticity at 222 nm for each coassembly as a function of temperature. Peptide concentrations are 25 μM in PBS (pH 7.0) + 2.5% HFIP (v/v). *T*_m,app_ values are reported as the average of three separate experiments ± the standard deviation.

c Δ*T*_m,app_ values were calculated relative to the wild-type value [Δ*T*_m,app_ = *T*_m,app_(wild type) −*T*_m,app_(variant)]. Negative Δ*T*_m,app_ values indicate destabilization.

Thermal denaturation experiments further revealed that unfolding of all HR1-HR2 assemblies occurs through a single, cooperative transition, as monitored by changes in ellipticity at 222 nm. The presence of a well-defined midpoint of unfolding (*T*_m,app_) for each complex indicates that disassembly of the coiled coil occurs in a highly cooperative manner, characteristic of well-ordered helical bundles. Importantly, no evidence of multistep unfolding or aggregation-induced signal distortion was observed under the experimental conditions, supporting direct comparison of thermal stabilities across variants. Together, these data establish that all examined HR2 mutations retain the capacity to form stable α-helical HR1-HR2 coiled-coil assemblies and that the observed differences in melting temperatures reflect genuine perturbations to postfusion core stability rather than peptide misfolding or misassembly.

This conclusion is further supported by crystallographic analysis, which reveals a minimal backbone deviation across all variants (Figure 4). We determined X-ray crystal structures of seven HR1-HR2 postfusion core assemblies, including the wild-type MeV fusion protein (PDB: 9Q4F) as well as neuropathogenic and hyperfusogenic variants L454W (PDB: 9ZUH), T461A (PDB: 9YG6), T461I (PDB: 9YD9), N462K (PDB: 9YFP), G464W (PDB: 9Y9O), and the triple mutant T461I/N462S/N465S (PDB: 9YAC). This series of structures provides a structural framework for interpreting how sequence perturbations modulate the assembly thermodynamics.

**Figure 4:**
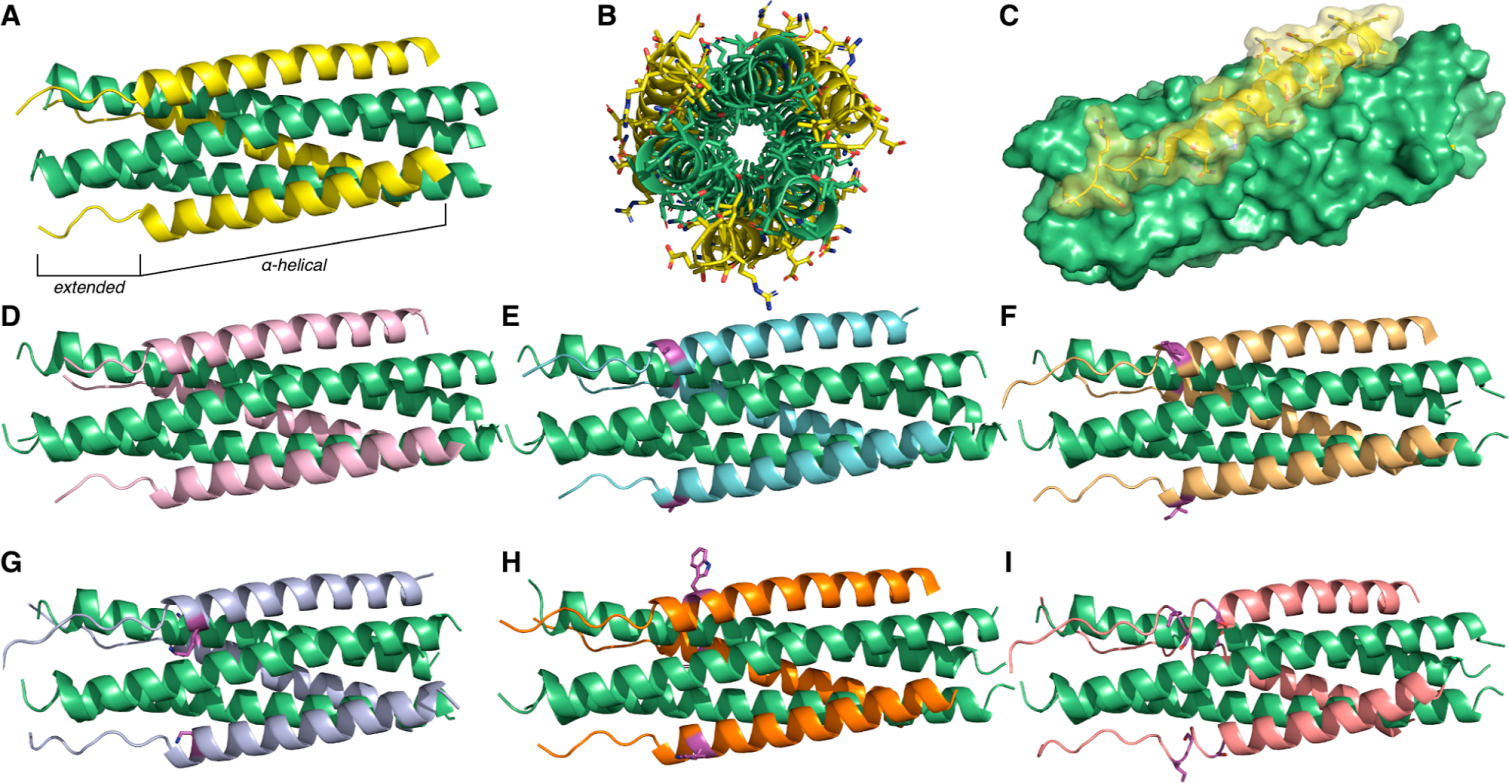
X-ray structures of MeV F postfusion core assemblies. (A) Cartoon representation of wild-type MeV F postfusion core, showing the canonical six-helix bundle (6HB) with three HR1 helices forming a central trimeric coiled-coil surrounded by three antiparallel HR2 helices. (B) Stick representation of the highlighting dense side chain packing at the HR1-HR2 interface. (C) Surface representation illustrating shape complementarity between HR1 and HR2. (D–I) Structures of HR1-HR2 assemblies containing HR2 mutations L454W, T461A, T461I, N462K, G464W, and T461I/N462S/N465S. Across all variants, the global 6HB architecture is preserved with minimal backbone deviation relative to the wild type. Structural differences are localized to side chain conformations and interaction networks near mutation sites.

All structures adopt the canonical class I fusion protein postfusion architecture, consisting of a central trimeric HR1 coiled-coil surrounded by three antiparallel HR2 helices to form a six-helix bundle. Each HR2 monomer adopts an extended structure at the N-terminus, which transitions to an α-helical secondary structure at Gly460. Alignment of variant structures onto the wild-type assembly yields backbone root-mean-square deviations of less than 0.3 Å (Table S4), indicating that mutations do not induce large-scale structural rearrangements. Quantitative analysis of the A_3_B_3_ assemblies revealed similar solvent-accessible surface areas and buried HR1-HR2 interface areas across all variants (Table S4), consistent with preservation of the overall six-helix bundle interface. Together, these metrics indicate that the global architecture of the postfusion core remains largely unchanged despite substantial differences in thermal stability. Instead, sequence variation is accommodated within a conserved architecture with differences localized to side chain conformations and interaction networks at specific positions along HR2.

Inspection of the structures reveals that residues within the HR1-HR2 interface engage in a network of hydrogen-bonding and packing interactions that stabilize the assembled core. These structures, therefore, provide direct molecular insight into how distinct mutations may perturb postfusion stability through localized disruption of steric compatibility and hydrogen-bonding networks without compromising overall six-helix bundle architecture.

### Hyperfusogenic HR2 Mutations Destabilize Postfusion Coiled-Coil Assembly

Having established that all HR1-HR2 variants form well-defined, cooperative α-helical assemblies, we next examined how mutations associated with hyperfusogenic phenotypes influence the thermodynamic stability of the postfusion 6HB. Apparent melting temperatures derived from CD thermal denaturation experiments (Table 1) provide a quantitative measure of postfusion core stability and enable direct comparison across variants under identical assembly conditions.

Unexpectedly, the majority of HR2 mutations examined resulted in a significant decrease in postfusion core stability relative to the wild-type HR1-HR2 assembly. Destabilization was observed for mutations located within both the extended N-terminal region and the α-helical segment of HR2. Most variants exhibited moderate destabilization (Δ*T*_m,app_ = −3 to −6 °C), although combinations of mutations produced more pronounced effects, with the T461I/N462S/N465S triple mutant displaying a substantial reduction in stability (Δ*T*_m,app_ = −10.3 °C). These changes correspond to meaningful energetic penalties for coiled-coil assembly. Statistical analysis of melting temperatures using one-way ANOVA followed by Dunnett’s multiple-comparison test (vs wild type) indicates that all variants, except T461I, differ significantly from the wild type assembly (*p* < 0.05), while T461I does not (*p* = 0.4781), consistent with its minimal effect on postfusion stability. Ultimately, these results indicate that even localized side chain substitutions can perturb 6HB stability.

In contrast, the only neuropathogenic or hyperfusogenic mutation tested that exhibited enhanced postfusion stability was E455G (Δ*T*_m,app_ = +4.5 °C). The rarity of stabilizing mutations underscores the intrinsic sensitivity of the HR1-HR2 interface to sequence variation and highlights the predominantly destabilizing influence of most hyperfusogenic substitutions. Collectively, these results demonstrate that hyperfusogenic mutations in MeV F do not generally enhance fusion by stabilizing the postfusion assembly. Rather, these data are consistent with models in which hyperfusogenicity arises from changes in prefusion stability or triggering efficiency. In this model, the postfusion assembly tolerates localized energetic penalties without loss of overall structural integrity or membrane fusion function.

### Mutations within the Extended N-Terminal Segment of HR2

Our crystal structure of the wild-type MeV fusion core revealed that the HR2 domain possesses an extended structure for the eight residues of the N-terminal segment, transitioning to an α-helical secondary structure for the remaining 28 residues. This nonhelical segment has been observed in related paramyxoviruses (e.g., HPIV3) and pneumoviruses (e.g., RSV) and has been shown to significantly influence both fusion core stability and fusogenic efficiency.^41,42^ To delineate how side chain properties influence postfusion assembly within the extended N-terminal segment of HR2, we examined established hyperfusogenic mutations at positions 454 and 455. This region, which precedes the well-defined HR2 helix, is expected to contribute to coiled-coil nucleation. However, unlike residues embedded within the helical interface, side chains in this region are expected to modulate assembly through steric complementarity and local flexibility.

Substitution of Leu454 with bulkier hydrophobic residues resulted in a consistent destabilization of the postfusion assembly. Both the L454W (neuropathogenic isolate) and L454M (laboratory-generated hyperfusogenic) variants exhibited reduced melting temperatures relative to the wild-type HR1-HR2 assembly, indicating that increased side-chain volume at this position imposes an energetic penalty on 6HB formation. Although Trp and Met differ in rigidity and polarity, both substitutions introduce steric bulk that may perturb local packing near the N-terminal boundary of the HR2 segment, highlighting the sensitivity of this region to side-chain complementarity. In contrast, substitution of Leu454 with alanine as a control experiment produced only a minimal change in stability, indicating that a reduced side chain steric footprint is tolerated at this position (see Supporting Information).

The compensatory E455G substitution provides further insight into the role of local flexibility and steric complementarity in the extended N-terminal segment of the HR2 domain. Introduction of glycine at position 455 significantly increased postfusion stability, consistent with the ability of glycine to relieve steric strain and accommodate conformational variability in the adjacent residues. When combined with the destabilizing L454W mutation, E455G partially restored stability, yielding a postfusion assembly with a melting temperature comparable to that of the wild-type assembly. This result suggests that enhanced backbone flexibility at position 455 can mitigate steric disruption introduced at adjacent residues, allowing more favorable packing and alignment of the extended HR2 segment within the coiled-coil assembly.

Structural analysis supports this observation, revealing that mutations at position 454 primarily affect local side-chain organization without altering the overall 6HB architecture. In the L454W variant, weak or poorly defined electron density for the bulky indole side chain indicates an increased conformational disorder within the extended N-terminal segment of HR2. Such local disorder is consistent with the observed thermodynamic destabilization and underscores the importance of steric complementarity at the coiled-coil nucleation sites. Collectively, these results characterize the extended N-terminal segment of HR2 as a region sensitive to increased steric bulk, which may be offset by increased flexibility through the introduction of glycine.

### Mutations within the α-Helical Segment of HR2

We next analyzed mutations within the α-helical segment of HR2 that directly contribute to stabilization of the postfusion 6HB through interhelical interactions. Structural analysis of the wild-type MeV postfusion core revealed that residues N462 and N465 are positioned at the HR1-HR2 interface, where their side chains engage in a cooperative network of hydrogen bonds that reinforce HR1-HR2 interaction and contribute to postfusion core stability (Figure 5a). Substitutions at these positions therefore provide a direct means of probing how disruption of interfacial hydrogen bonding influences coiled-coil assembly thermodynamics.

**Figure 5:**
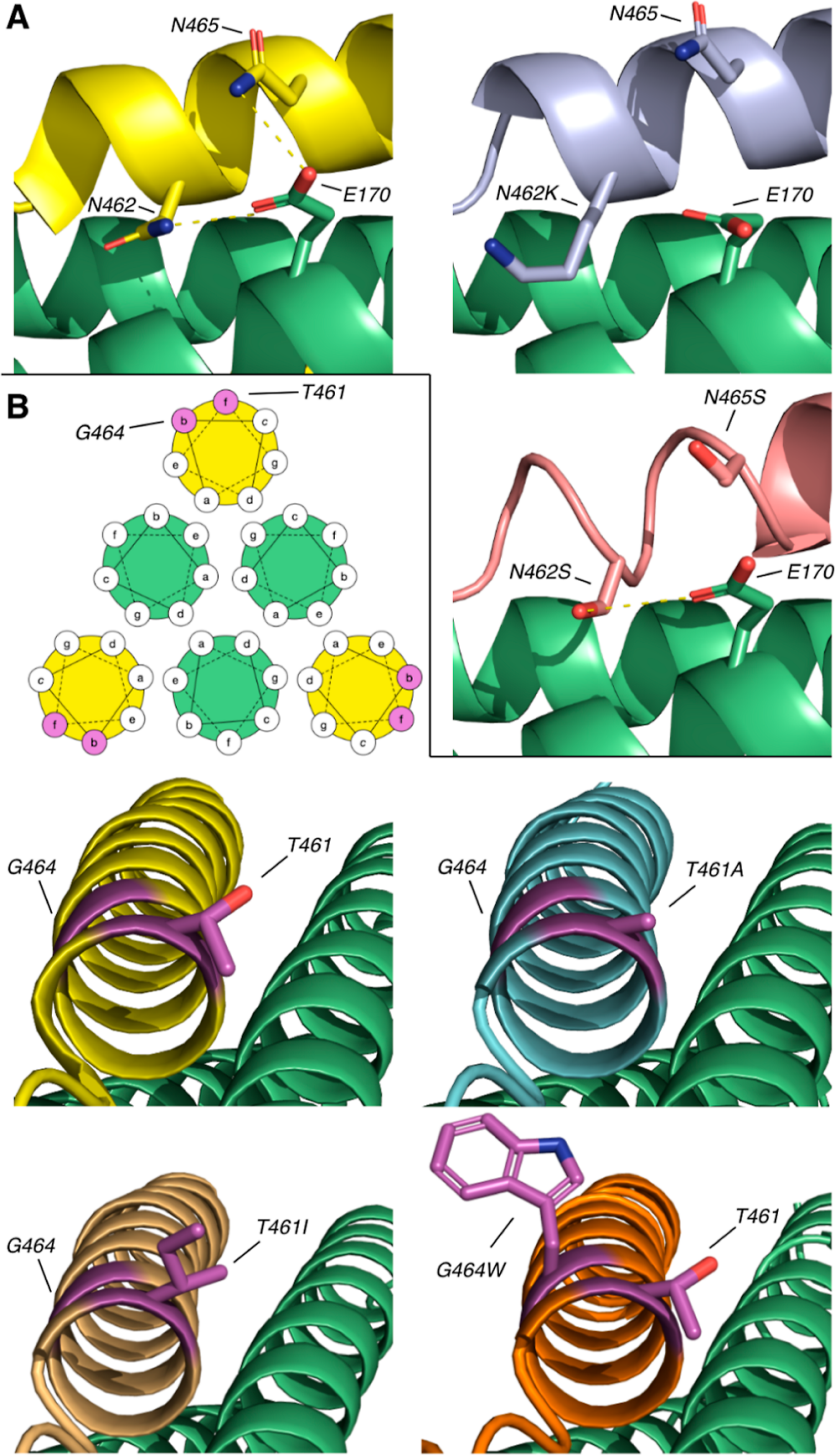
Structural determinants of HR2-mediated stability within the MeV postfusion six-helix bundle. (A) Close-up views of the HR1-HR2 interface highlighting stabilizing hydrogen-bonding interactions in the wild-type postfusion core and their disruption in destabilized variants. In the wild-type structure, HR2 residues N462 and N465 (yellow) form hydrogen bonds with HR1 residue E170 (green). In the N462 K (purple) and T461I/N462S/N465S (salmon) variants, this interaction is disrupted. (B) Structural context of solvent-exposed HR2 residues T461 and G464. A heptad-repeat schematic shows that T461 and G464 occupy *f* and *b* positions, respectively, orienting their side chains away from the HR1 interface. Corresponding zoomed-in views of the wild-type (yellow), T461A (cyan), T461I (light orange), and G464W (dark orange) structures support this orientation.

Single substitutions at positions 462 or 465 resulted in pronounced destabilization of the postfusion assembly. Replacement of Asn462 with serine or lysine led to substantial destabilization, consistent with loss of a stabilizing hydrogen bond between the side chain amide of N462 and the adjacent HR1 residue E170. Crystallographic analysis confirms that neither the shorter serine side chain of N462S nor the extended, conformationally flexible lysine side chain of N462K retains the interhelical interaction observed in the wild-type structure between N462 and E170. Similarly, substitution of Asn465 with serine eliminates multiple hydrogen bonding contacts that stabilize both the HR2 α-helix and its interface with HR1, resulting in a significant energetic penalty. These observations underscore the importance of side chain-mediated hydrogen bonding in maintaining the integrity of the α-helical HR1-HR2 interface.

The cumulative impact of disrupting interhelical contacts is most evident in the T461I/N462S/N465S triple mutant, which displays the largest reduction in postfusion core stability observed in this study. This result suggests cooperativity within the hydrogen bond network at the HR1-HR2 interface. Importantly, crystallographic analysis demonstrates that even in this significantly destabilized variant, the global 6HB architecture remains intact with backbone alignment revealing minimal deviation from the wild-type structure. Thus, the energetic penalty arises from localized loss of stabilizing interactions rather than from a large-scale structural rearrangement.

Although residue T461 is positioned within the HR2 helical segment adjacent to residues N462 and N465S discussed above, structural analysis shows that this residue is solvent-exposed and does not participate directly in the interhelical hydrogen bond network. Accordingly, substitutions at this position were considered separately. Taken together, the results presented here establish that stability of the HR2 helical interface is governed by a network of cooperative hydrogen bonds and hydrophobic packing interactions and that disruption of these interactions produces disproportionately large energetic penalties while preserving the overall 6HB architecture.

### Mutations at Solvent-Exposed α-Helical Regions of HR2

In contrast to residues that stabilize the HR1-HR2 interface through direct hydrogen bonding or packing interactions, solvent-exposed positions within the HR2 helix are expected to play a less prominent role in assembly stability. Residues T461 and G464 occupy such positions, oriented toward solvent and away from the HR1-HR2 interface (Figure 5b). Substitutions at these sites are, therefore, expected to exhibit comparatively smaller effects.

Replacement of Thr461 with isoleucine did not significantly alter postfusion stability relative to that of the wild-type assembly. In contrast, substitution with alanine produced modest but significant destabilization. These results suggest that Thr461 may contribute favorable solvation or backbone-stabilizing interactions that are lost upon replacement with alanine, whereas the isoleucine substitution is comparatively well tolerated.

Mutations at G464, another solvent-exposed position, reveal an interplay between α-helix propensity and conformational entropy at this site. Substitution of G464 with alanine, a mutation not observed in MeV variants but examined as a control, increased postfusion stability by 5.6 °C (see Supporting Information), consistent with the exceptionally low helix propensity and high conformational entropy of glycine relative to alanine.^46^ In this context, replacement of glycine with a small, helix-promoting residue may reduce the entropic cost of helix formation without introducing steric or packing frustration, thereby stabilizing coiled-coil assembly. In contrast, substitution with tryptophan (G464W) resulted in modest destabilization of the postfusion assembly. This destabilization may be attributed to the introduction of a bulky, conformationally constrained aromatic side chain that creates local steric and solvation incompatibility despite being at a solvent-exposed position.

These results demonstrate that solvent-exposed positions within the HR2 helix modulate postfusion assembly stability through a balance of intrinsic helix propensity, conformational entropy, and solvation effects. Unlike interfacial residues, where disruption of hydrogen bond networks produces large energetic penalties, substitutions at solvent-exposed sites exert more nuanced effects that nevertheless measurably influence 6HB stability. These findings underscore the importance of residue-specific context in coiled-coil assemblies and highlight how classical principles of peptide helix formation intersect with higher-order assembly energetics in viral fusion cores.

## Conclusions

In this study, we combined peptide biophysics and high-resolution structural analysis to define how targeted mutations within the HR2 domain of the MeV fusion glycoprotein modulate postfusion core assembly. By isolating HR1-HR2 interactions in a chemically defined peptide system, we have demonstrated that mutations associated with neuropathogenic and hyperfusogenic phenotypes consistently decrease postfusion core stability despite preserving the global 6HB architecture. These findings demonstrate that hyperfusogenic mutations are not associated with stabilization of the postfusion state, even when the global postfusion 6HB architecture remains intact.

While the present study focuses on the thermodynamic stability of the postfusion six-helix bundle, hyperfusogenic phenotypes are also governed by the kinetics of the prefusion-to-postfusion transition. In this context, enhanced fusion activity does not require stabilization of the postfusion state but may instead arise from a reduction in the activation barrier for fusion. Such effects could result from destabilization of the prefusion conformation, as has been reported for several measles virus variants, or from altered interactions with partner proteins, such as the hemagglutinin (H) protein, which regulates triggering of F protein refolding. The observation that the mutations examined here consistently destabilize the postfusion assembly therefore argues against a mechanism based on increased thermodynamic stabilization of the fusion core and instead supports models in which hyperfusogenicity arises from changes in prefusion stability or triggering efficiency.

Prior thermodynamic studies of class I viral fusion proteins have established that stability of coiled-coil assemblies can influence binding interactions, inhibitor design, and, in some cases, fusion efficiency. In particular, studies of HIV-1 gp41 ^47–53^ and SARS-CoV-1/2 spike^38,54–56^ have shown that postfusion six-helix bundle stability is influenced by hydrophobic packing, hydrogen bonding, and other cooperative interactions within the HR1-HR2 interface. These studies established important thermodynamic principles governing class I fusion protein assemblies and highlighted that relatively localized perturbations can substantially alter the energetic properties of postfusion complexes. The present results are consistent with these general principles but reveal an important distinction: hyperfusogenic mutations in MeV do not enhance fusion through stabilization of the postfusion assembly and instead frequently destabilize the six-helix bundle. These observations suggest that the relationship between postfusion stability and fusion efficiency may be context-dependent and influenced by the balance between prefusion stability, activation kinetics, and postfusion energetics.

Mechanistically, our results reveal that postfusion assembly stability is governed by distinct, context-dependent chemical factors. Steric complementarity and local flexibility dominate within the extended N-terminal segment of HR2. Cooperative interhelical hydrogen-bonding networks are important for stabilizing the HR1-HR2 interface. Intrinsic helix propensity and solvation effects control the solvent-exposed positions within the HR2 helix. Disruption of interfacial hydrogen bonds produces disproportionately large energetic penalties, whereas perturbations at solvent-facing sites exert more-nuanced effects. Together, these observations illustrate how localized side-chain chemistry can reshape coiled-coil energetics without altering higher-order structure.

More broadly, this work demonstrates that hyperfusogenicity can occur independently of postfusion thermodynamic stabilization. From a peptide chemistry perspective, these findings provide a quantitative framework for understanding sequence-structure–stability relationships in coiled-coil assemblies and offer guidance for the rational design of HR1- and HR2-derived peptide inhibitors that exploit the energetic vulnerabilities of viral fusion proteins.

## Supplementary Material



## Notes

Experimental procedures, characterization data, CD spectra, X-ray data collection, and refinement statistics (PDF)

## Abbreviations

CD: circular dichroism
MeV: measles virus
6HB: six-helix bundle
