# Supplementary material for: Hyperfusogenic Mutations Destabilize the Postfusion Six-Helix Bundle of the Measles Virus Fusion Glycoprotein
Source: Biochemistry. 2026 Jul 22;65(15):2350–8. doi: 10.1021/acs.biochem.6c00182 (PMC13445589; doi:10.1021/acs.biochem.6c00182)
Supplement: Supplementary file 1 [file bi6c00182_si_001.pdf]

Supporting Information

Hyperfusogenic Mutations Destabilize the Postfusion Six-Helix Bundle of the Measles Virus Fusion Glycoprotein

Nisansala Vithanage, Victor K. Outlaw*

Department of Chemistry, University of Missouri, Columbia, Missouri, 65203, United States

* Corresponding Author. Address correspondence to: Victor K. Outlaw, victoroutlaw@missouri.edu

Table of Contents

Peptide Synthesis and Purification *3*

General Procedure **3**

Circular Dichroism Experiments *24*

Instrumentation **24**

Wavelength Scan **24**

Thermal Denaturation **24**

X-ray Crystallography *32*

Co-crystallization **32**

X-ray Data Collection, Processing, Structure Solution, and Refinement **34**

Classification *42*

References *43*

Peptide Synthesis and Purification

General Procedure

Peptides were prepared by manual or microwave-assisted solid-phase peptide synthesis (SPPS) using Rink amide ProTide low-loading resin (CEM). All Fmoc-protected amino acids were purchased from Chem-Impex and included: Asp(*t*-Bu ester), Glu(*t*-Bu ester), Lys(Boc), Asn(trityl), Gln(trityl), Arg(Pbf), Ser(*t*-Bu ether), Thr(*t*-Bu ether), Trp(Boc), and Tyr(*t*-Bu ether).

Manual SPPS

The resin was pre-swelled with DMF in a polypropylene fritted syringe (Torviq), then drained and washed with 2 resin volumes of DMF. Peptide elongation was performed by successive coupling and deprotection reactions. Coupling reactions were performed using solutions comprised of 4 equivalents Fmoc-protected amino acid, 4 equivalents of 1-[bis(dimethylamino)methylene]-1H-1,2,3-triazolo[4,5-b]pyridinium 3-oxide hexafluorophosphate (HATU, Chem-Impex), and 8 equivalents of diisopropylethylamine (DIEA, Oakwood) in dimethylformamide (DMF, Millipore-Sigma) at a final concentration of 100 mM Fmoc-amino acid. Coupling reactions were stirred for 20 minutes at room temperature. Double-coupling procedure was applied for Ile, Leu and Val residues to ensure efficient incorporation. The resin was washed with 1–2 resin volumes of DMF. Deprotection reactions were performed by addition of 20% (v/v) piperidine (Sigma-Aldrich) in DMF. The deprotection reactions were stirred for 10 minutes at room temperature. The resin was washed with 3–4 resin volumes of DMF.

Microwave-assisted SPPS

Microwave-assisted solid phase peptide synthesis was performed on a CEM Liberty Blue automated microwave peptide synthesizer. The resin was pre-swelled with DMF, then drained and washed with DMF. Peptide elongation was performed by successive coupling and deprotection reactions. Coupling reactions were performed using 4 equivalents Fmoc-protected amino acid, 4 equivalent of diisopropyl carbodiimide (DIC, Chem-Impex), and 8 equivalents of Oxyma (Chem-Impex). Coupling reactions were heated to 90 °C over 30 seconds and maintained at that temperature for 100 seconds, followed by draining and washing with DMF. Double-coupling strategy was employed for Arg, Ile, Leu, and Val residues. Deprotection reactions were performed by addition of 20% (v/v) piperidine in DMF. The deprotection reactions were heated to 90 °C over 30 seconds and maintained at that temperature for 45 seconds, followed by draining and washing with DMF.

Capping and Cleavage

Following the final deprotection step, the N-terminus of the peptide was capped by stirring the resin in 8:2:1 DMF:DIEA:Ac_2O at 23 ºC for 20 minutes. The resin was washed with DMF (3 × 25 mL) and DCM (3 × 25 mL), and peptide cleavage from resin was achieved by mixing with a solution of 94% trifluoroacetic acid (TFA, Sigma-Aldrich), 2.5% H_2O, and 2.5% triisopropylsilane (TIS, Sigma-Aldrich) for 2.5 hours. For the HR2-L454M peptide, cleavage was performed with a cocktail with containing 94% TFA, 2.5% ethanedithiol (EDT, Sigma-Aldrich), 2.5% H_2O, and 2.5% TIS. The resulting solution was filtered through the fritted syringe, the remaining resin was washed with 2–3 column volumes of TFA, and the combined extracts were concentrated under N_2. The peptide was precipitated by addition of cold diethyl ether, pelleted by centrifugation, and dried under N_2 to afford the crude product as a powder.

Purification and Characterization

Purification of peptides was achieved by reversed-phase HPLC on an Agilent Infinity II preparative HPLC system equipped with an Eclipse XDB-C18 column (21.2 × 250 mm with 7 µm pore size) using a gradient of degassed water (+0.1% TFA) and acetonitrile (+0.1% TFA). The identity of each peptide was confirmed by mass analysis using a Shimadzu MALDI-8020 mass spectrometer. Several peptides exhibit a minor peak eluting ~1–2 minutes after the major product peak in analytical HPLC traces. MALDI-TOF mass spectrometry confirms that this feature corresponds to the same exact mass as the desired peptide. Reinjection of the isolated major peak reproduces the same minor feature, suggesting that it arises from aggregation of these highly hydrophobic peptides under reversed-phase conditions rather than from chemical impurities. Peptide purity >95% was established by peak area integration using reversed-phase HPLC on an Agilent Infinity II analytical HPLC system equipped with an Eclipse XDB-C18 column (4.5 × 250 mm with 5 µm pore size) using a 10–95% H_2O/MeCN (+0.1% TFA) gradient over 6 minutes.

MeV F HR1 domain (MeV F HR1-wt)

Sequence

Ac-LNSQAIDNLRASLETTNQAIEAIRQAGQETILAVQGVQDYINNELIPS-NH_2

Calculated monoisotopic $[M+H]^+$: 5262.8

Calculated monoisotopic $[M+2H]^+$: 2631.4

Observed $[M+H]^+$: 5263.2

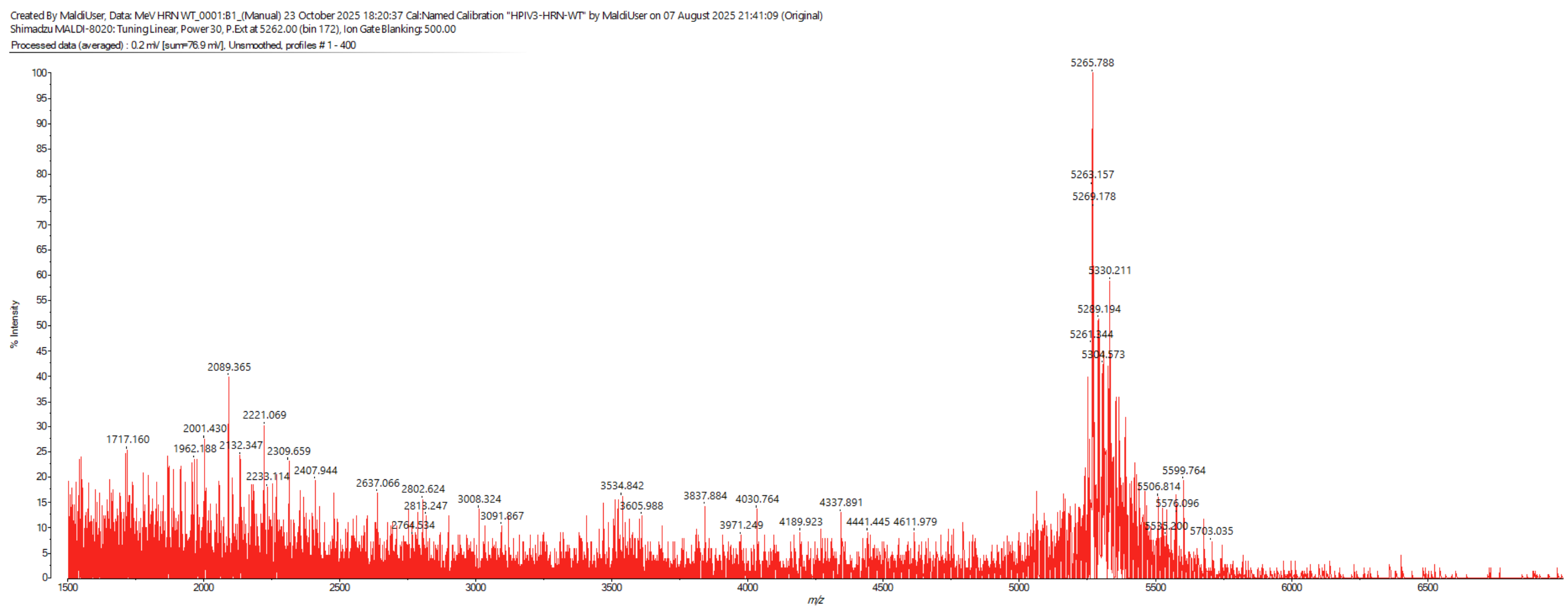

Fig. S1. MALDI-TOF MS spectrum of HR1-wt.

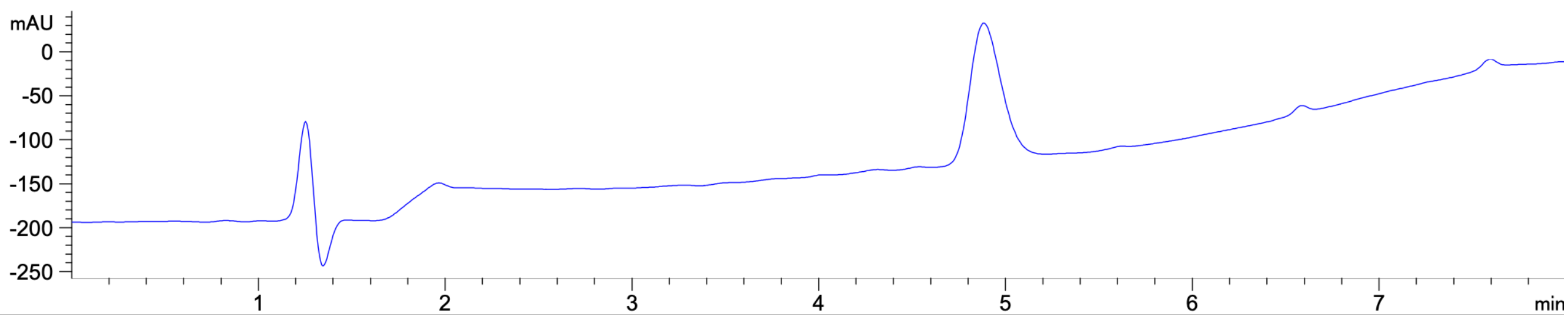

Fig. S2. Analytical HPLC chromatogram of purified HR1-wt (purity > 95%).

MeV F HR2 domain (MeV F HR2-wt)

Sequence

Ac-ISLERLDVGTNLGNAIAKLEDAKELLESSDQILRS(nLe)-NH_2

Calculated monoisotopic $[M+H]^+$: 3964.1

Calculated monoisotopic $[M+2H]^+$: 1982.1

Observed $[M+H]^+$: 3964.1

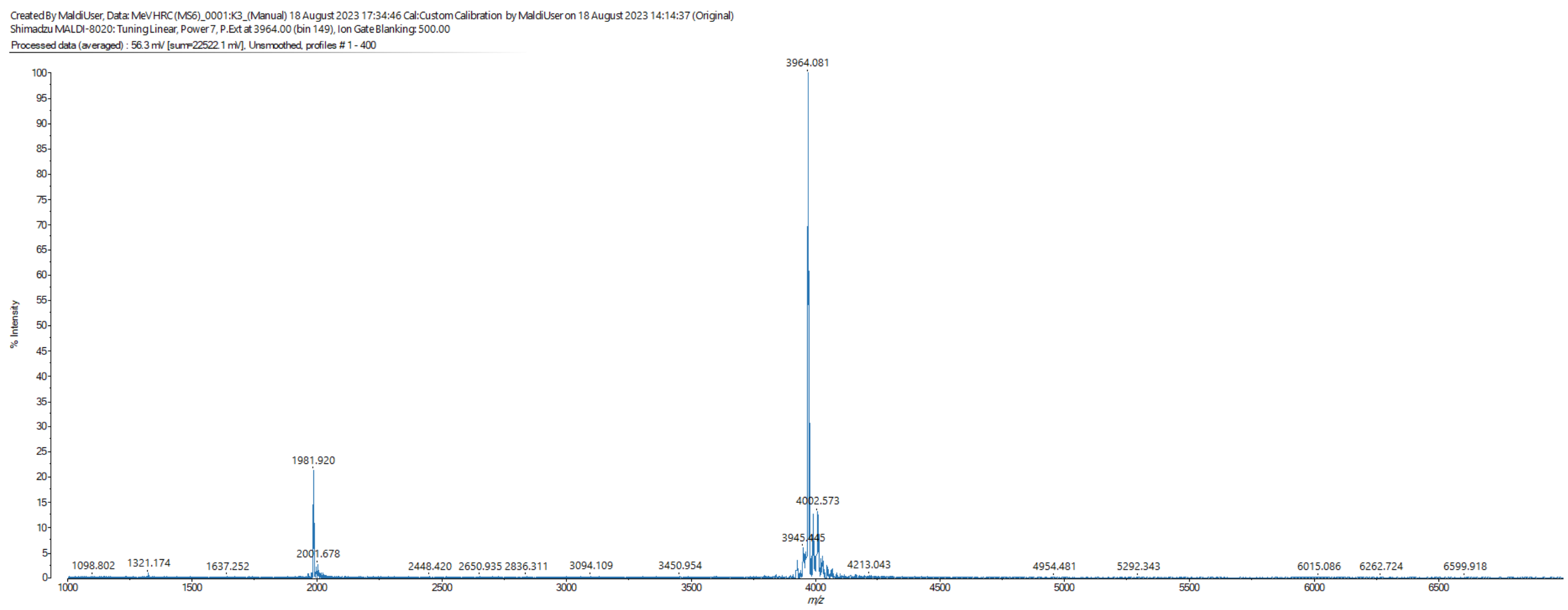

Fig. S3. MALDI-TOF MS spectrum of HR2-wt.

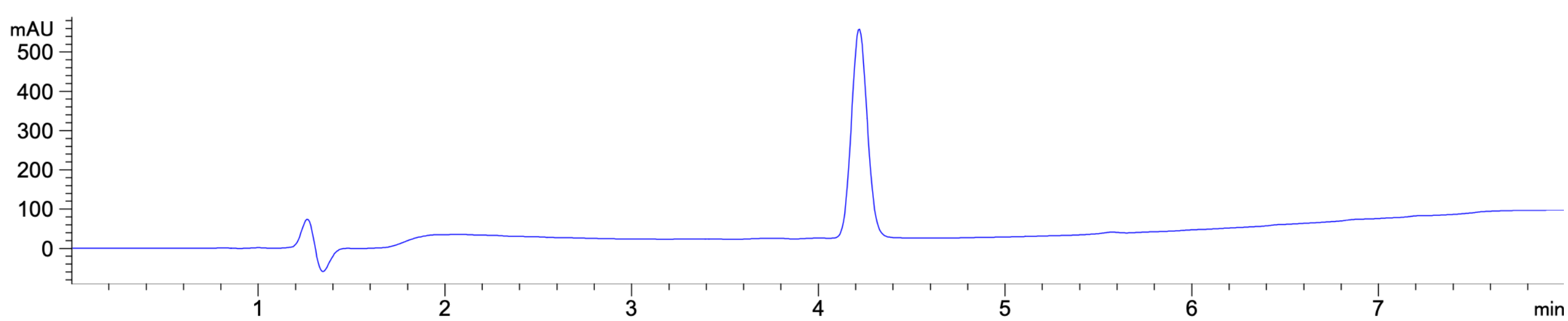

Fig. S4. Analytical HPLC chromatogram of purified HR2-wt (purity > 95%).

MeV F HR2-L454W)

Sequence

Ac-ISWERLDVGTNLGNAIAKLEDAKELLESSDQILRS(nLe)-NH_2

Calculated monoisotopic $[M+H]^+$: 4037.1

Calculated monoisotopic $[M+2H]^+$: 2018.6

Observed $[M+H]^+$: 4037.1

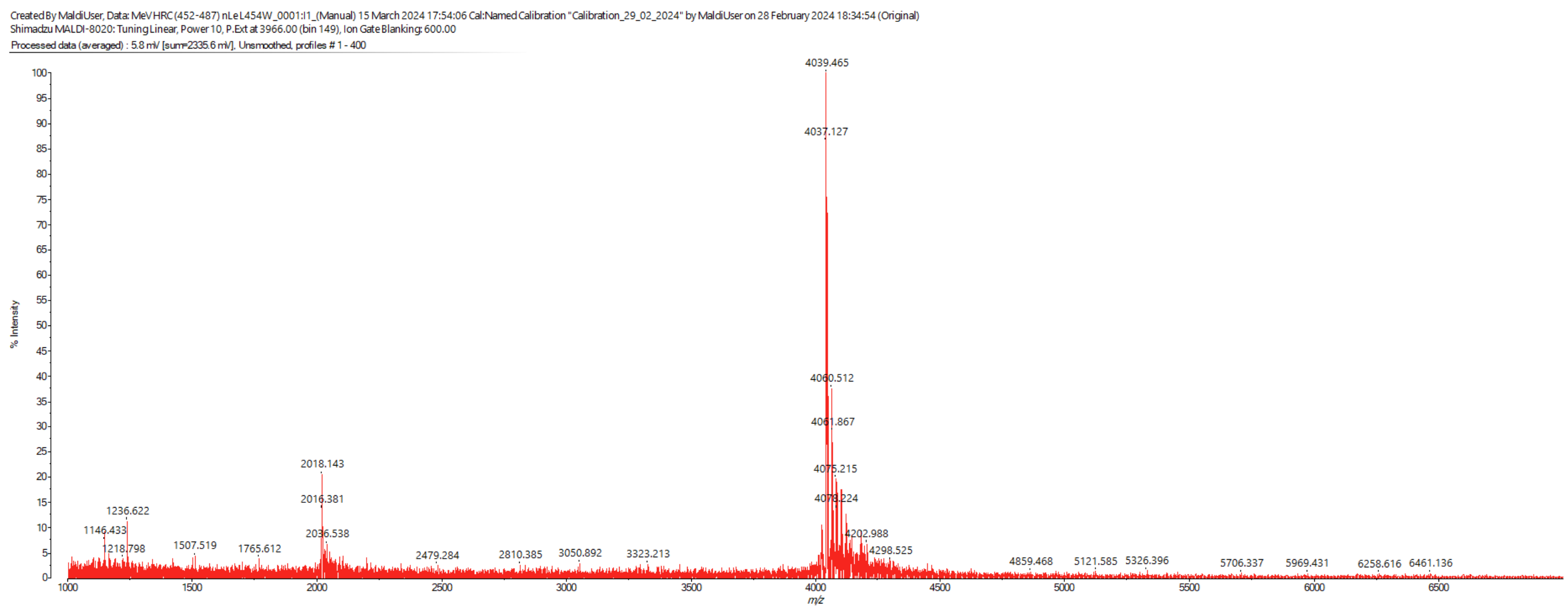

Fig. S5. MALDI-TOF MS spectrum of HR2-L454W.

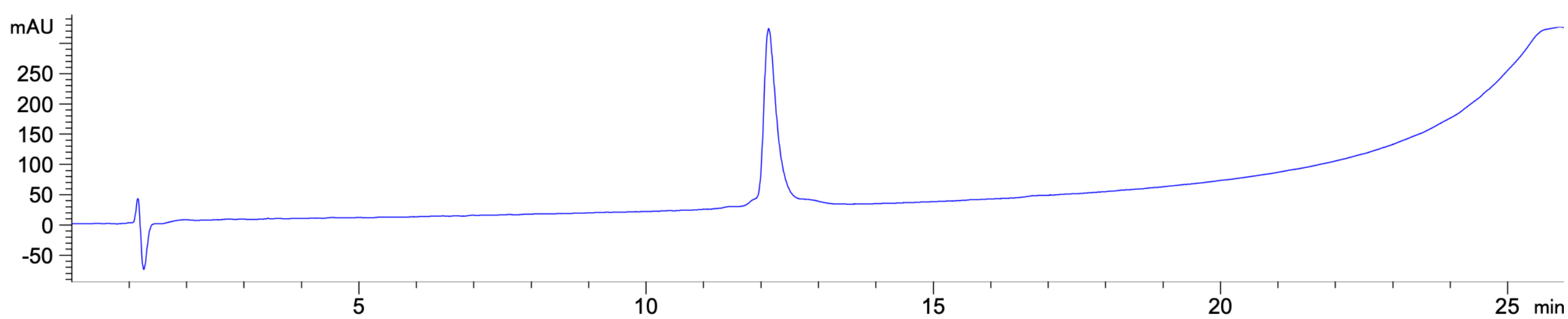

Fig. S6. Analytical HPLC chromatogram of purified HR2-L454W (purity > 95%).

MeV F HR2-L454M

Sequence

Ac-ISMERLDVGTNLGNAIAKLEDAKELLESSDQILRS(nLe)-NH_2

Calculated monoisotopic $[M+H]^+$: 3982.1

Calculated monoisotopic $[M+2H]^+$: 1991.0

Observed $[M+H]^+$: 3985.7

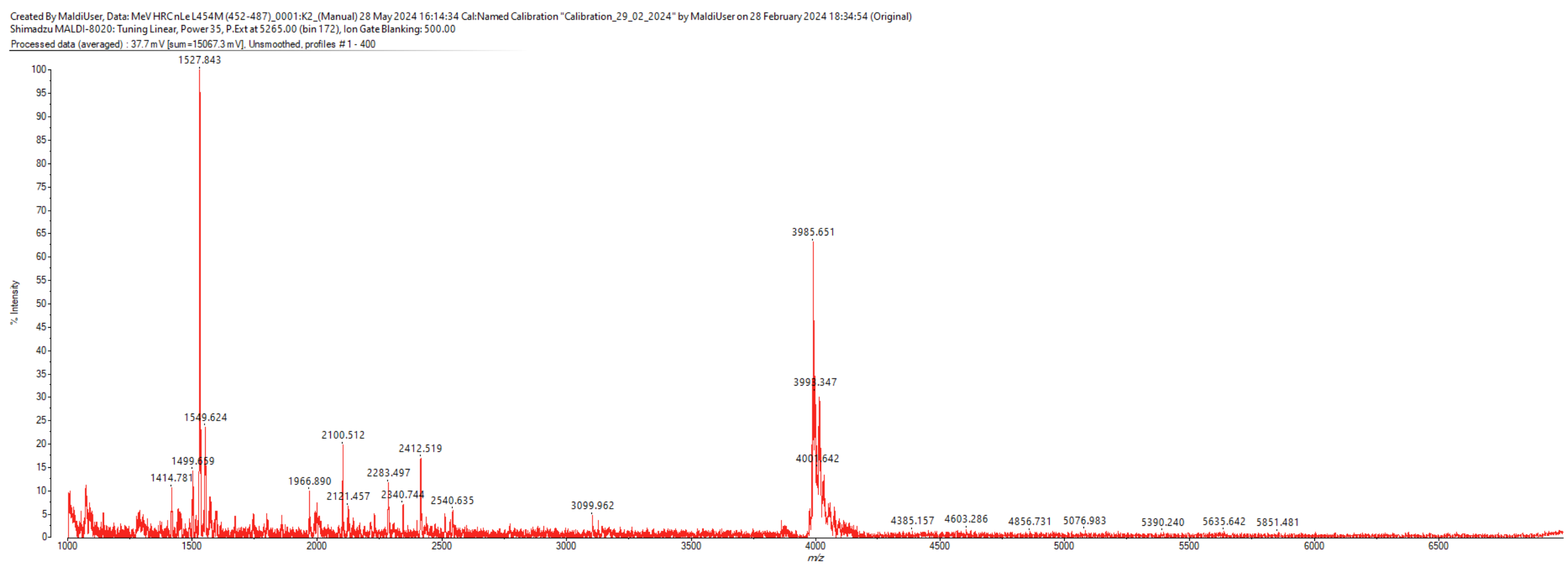

Fig. S7. MALDI-TOF MS spectrum of HR2-L454M.

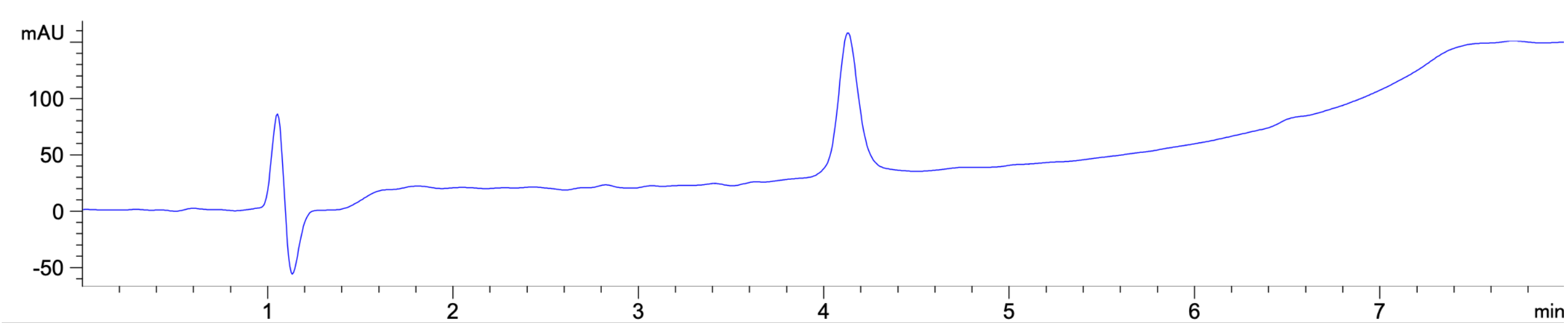

Fig. S8. Analytical HPLC chromatogram of purified HR2-L454M (purity > 95%).

MeV F HR2-L454A

Sequence

Ac-ISAERLDVGTNLGNAIAKLEDAKELLESSDQILRS(nLe)-NH_2

Calculated monoisotopic $[M+H]^+$: 3922.1

Calculated monoisotopic $[M+2H]^+$: 1961.0

Observed $[M+H]^+$: 3923.9

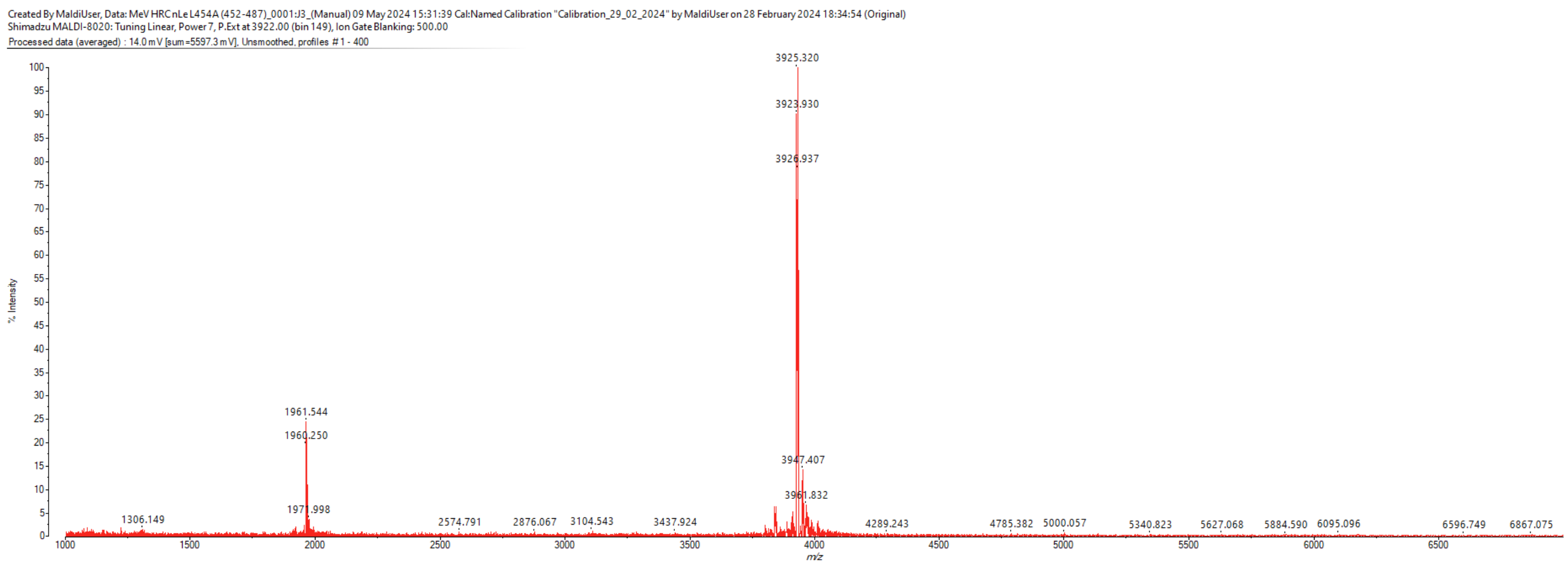

Fig. S9. MALDI-TOF MS spectrum of HR2-L454A.

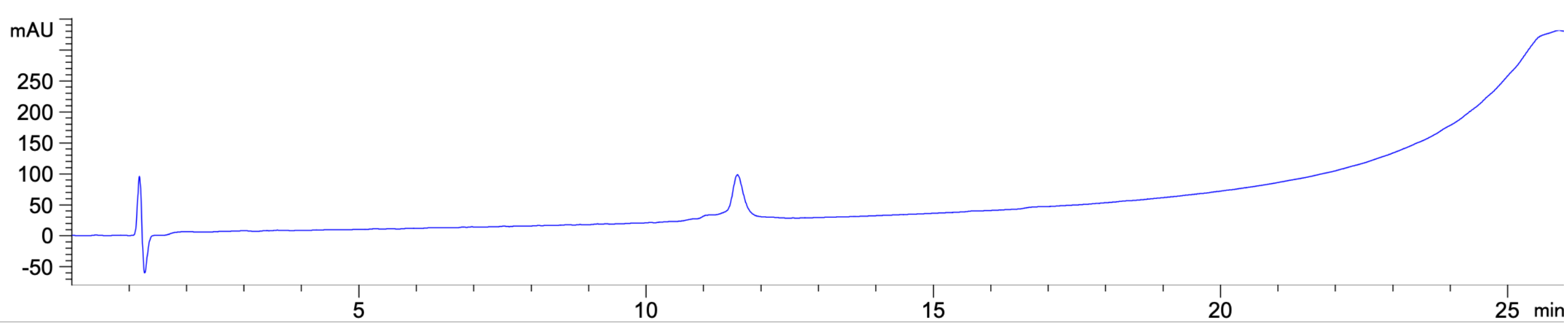

Fig. S10. Analytical HPLC chromatogram of purified HR2-L454A (purity > 95%).

MeV F HR2- E455G

Sequence

Ac-ISLGRLDVGTNLGNAIAKLEDAKELLESSDQILRS(nLe)-NH_2

Calculated monoisotopic $[M+H]^+$: 3892.1

Calculated monoisotopic $[M+2H]^+$: 1946.1

Observed $[M+H]^+$: 3894.4

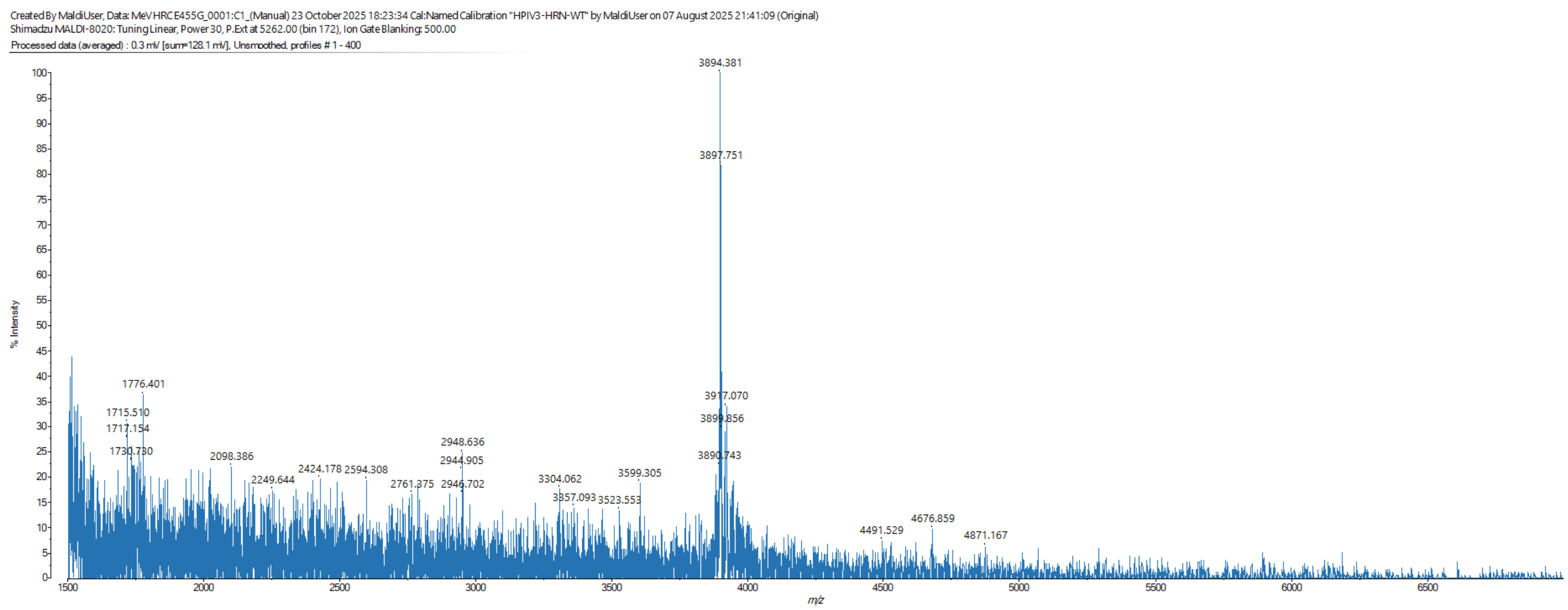

Fig. S11. MALDI-TOF MS spectrum of HR2-E455G.

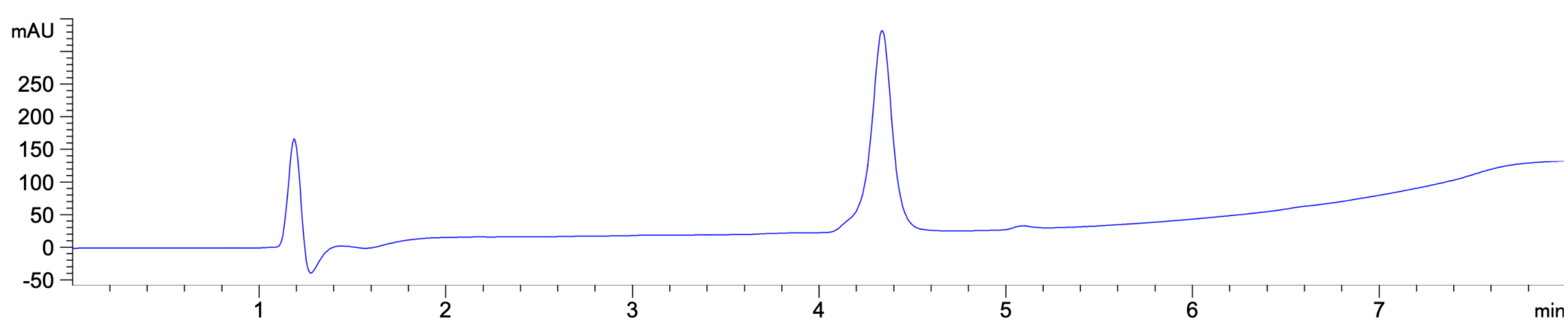

Fig. S12. Analytical HPLC chromatogram of purified HR2-E455G (purity > 95%).

MeV F HR2-L454W/E455G

Sequence

Ac-ISWGRLDVGTNLGNAIAKLEDAKELLESSDQILRS(nLe)-NH_2

Calculated monoisotopic $[M+H]^+$: 3965.1

Calculated monoisotopic $[M+2H]^+$: 1983.6

Observed $[M+2H]^+$: 1982.5

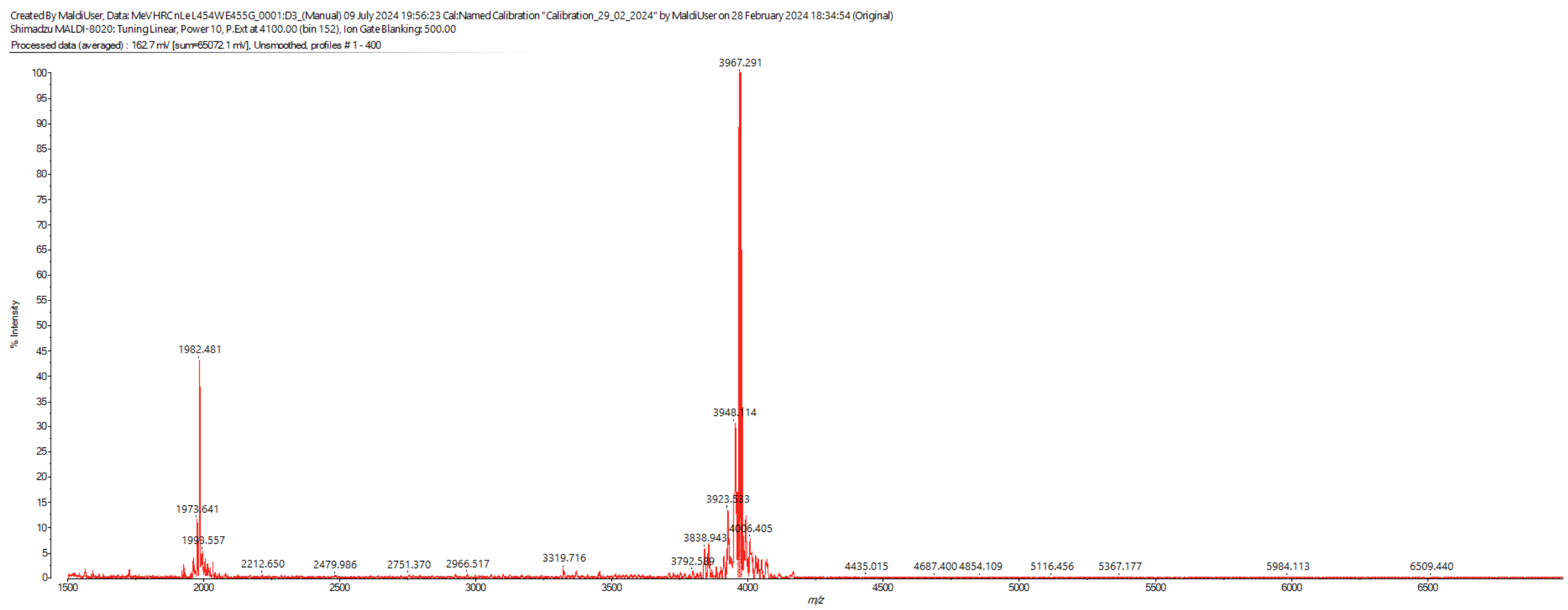

Fig. S13. MALDI-TOF MS spectrum of HR2-L454W/E455G.

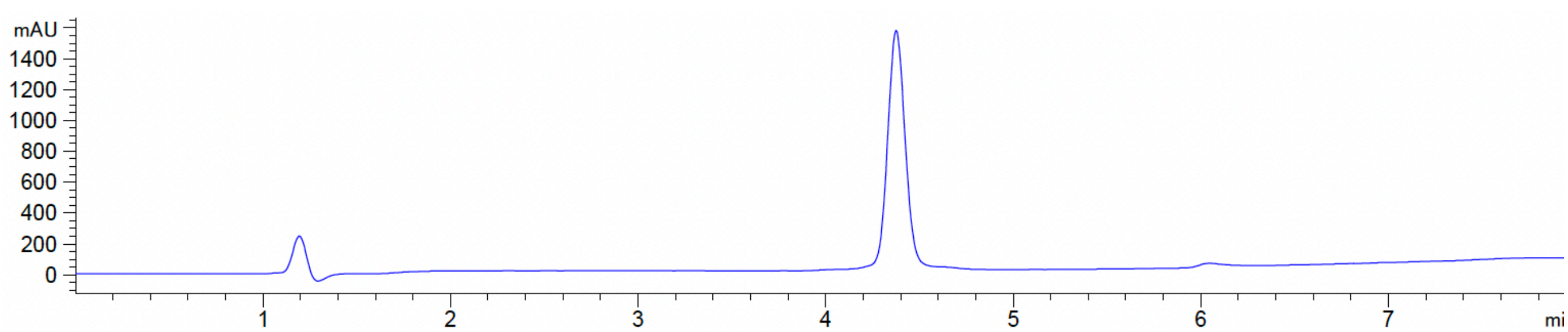

Fig. S14. Analytical HPLC chromatogram of purified HR2-L454W/E455G (purity > 95%).

MeV F HR2-L454A/E455G

Sequence

Ac-ISAGRLDVGTNLGNAIAKLEDAKELLESSDQILRS(nLe)-NH_2

Calculated monoisotopic $[M+H]^+$: 3850.1

Calculated monoisotopic $[M+2H]^+$: 1926.0

Observed $[M+2H]^+$: 1927.6

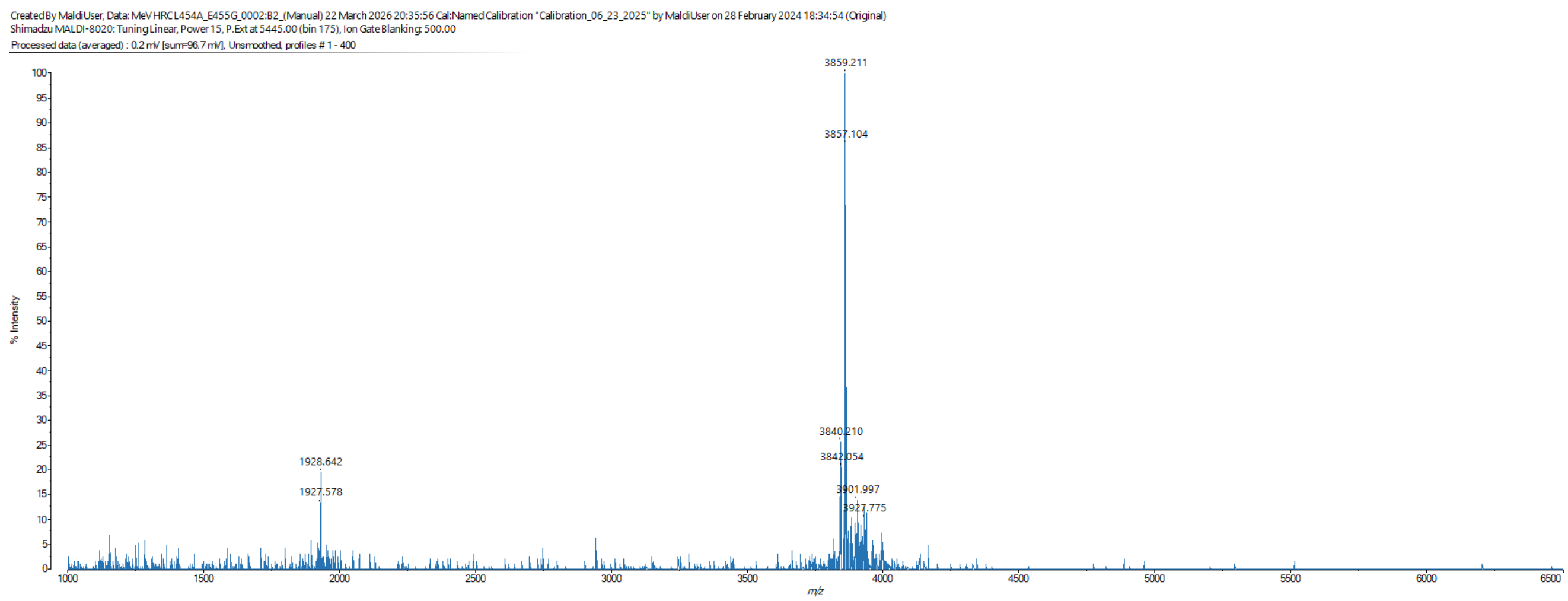

Fig. S15. MALDI-TOF MS spectrum of HR2-L454A/E455G.

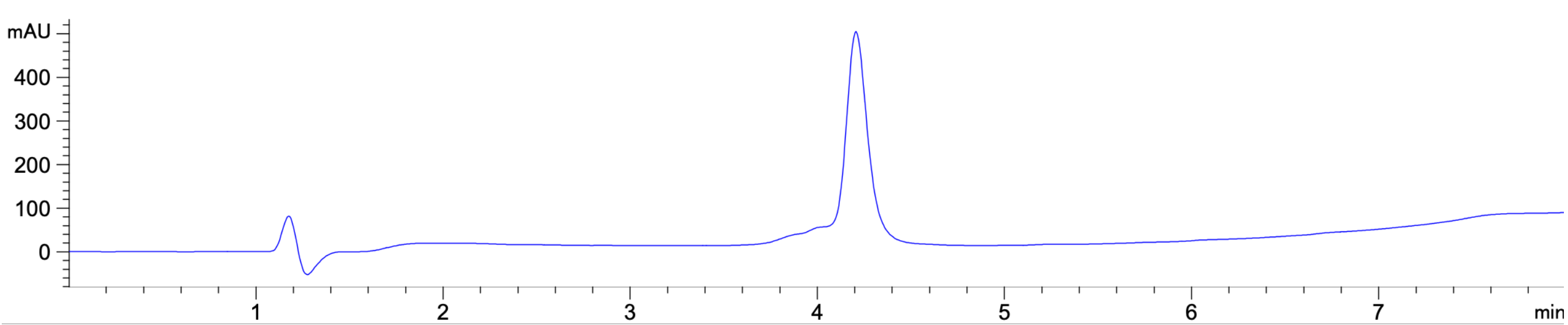

Fig. S16. Analytical HPLC chromatogram of purified HR2-L454A/E455G (purity > 95%).

MeV F HR2-T461I

Sequence

Ac-ISLERLDVGINLGNAIAKLEDAKELLESSDQILRS(nLe)-NH_2

Calculated monoisotopic $[M+H]^+$: 3976.2

Calculated monoisotopic $[M+2H]^+$: 1988.1

Observed $[M+H]^+$: 3977.9

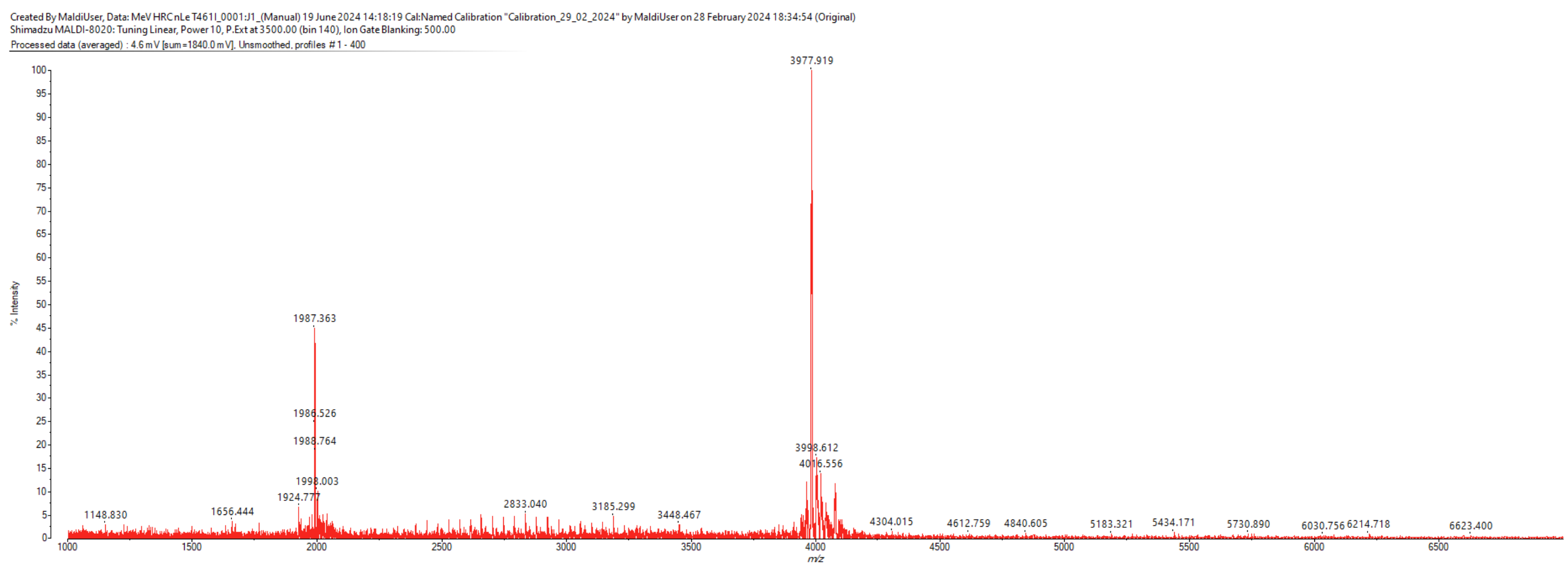

Fig. S17. MALDI-TOF MS spectrum of HR2-T461I.

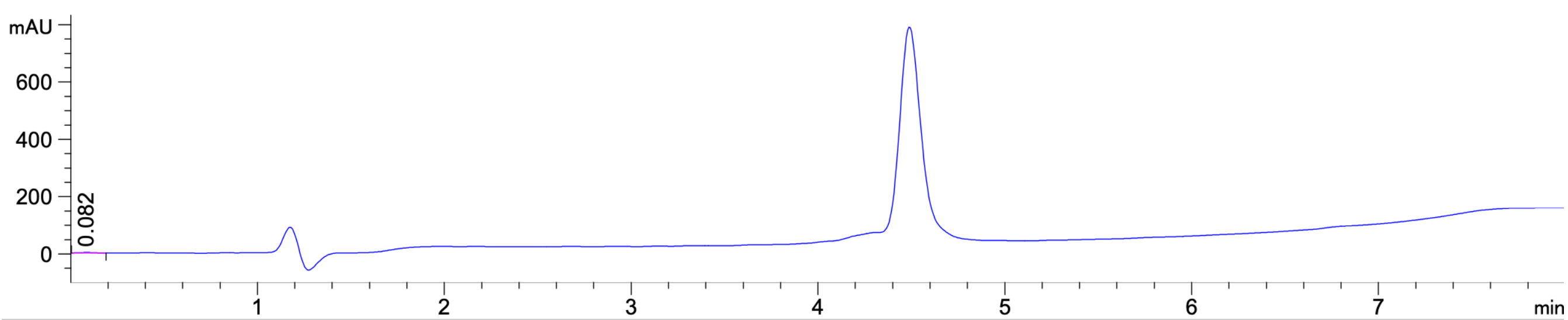

Fig. S18. Analytical HPLC chromatogram of purified HR2-T461I (purity > 95%).

MeV F HR2-T461A

Ac-ISLERLDVGANLGNAIAKLEDAKELLESSDQILRS(nLe)-NH_2

Calculated monoisotopic $[M+H]^+$: 3934.1

Calculated monoisotopic $[M+2H]^+$: 1967.1

Observed $[M+H]^+$: 3934.2

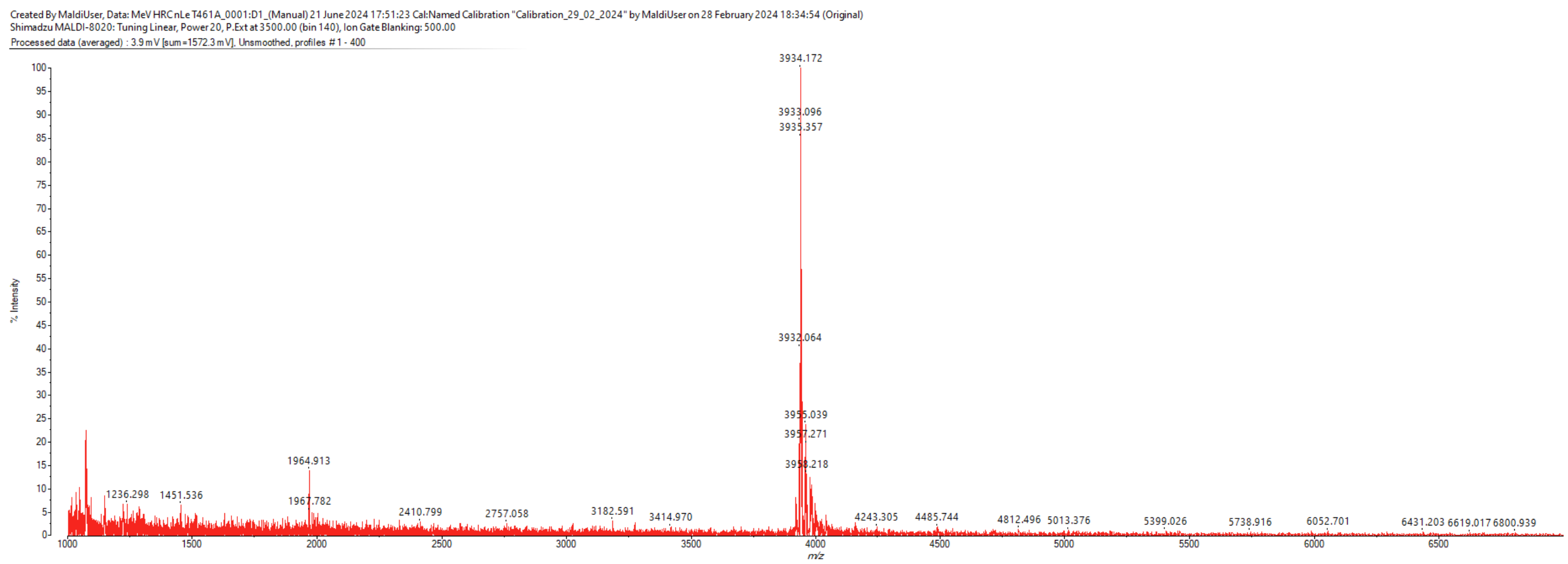

Fig. S19. MALDI-TOF MS spectrum of HR2-T461A.

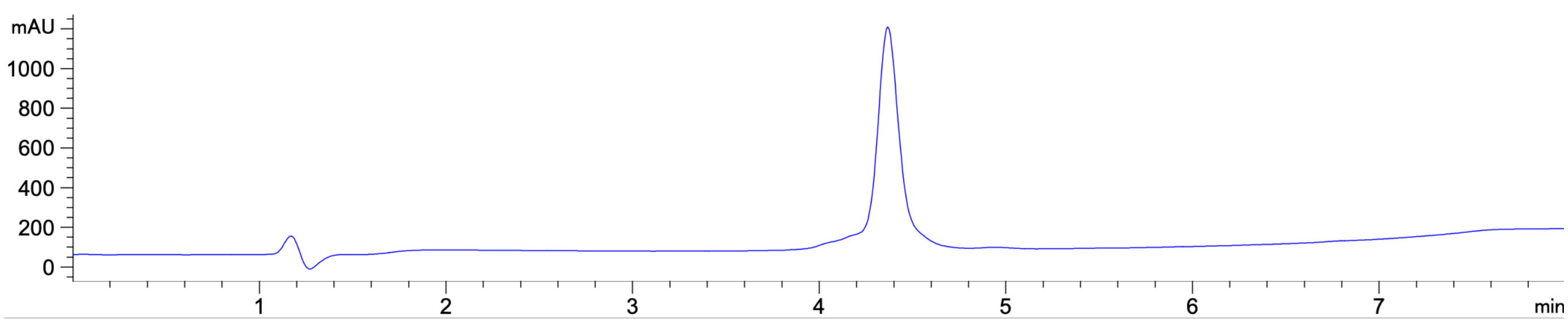

Fig. S20. Analytical HPLC chromatogram of purified HR2-T461A (purity > 95%).

MeV F HR2-N462K

Sequence

Ac-ISLERLDVGT**K**LGNAIAKLEDAKELLESSDQILRS(nLe)-NH_2

Calculated monoisotopic $[M+H]^+$: 3978.2

Calculated monoisotopic $[M+2H]^+$: 1989.1

Observed $[M+H]^+$: 3978.7

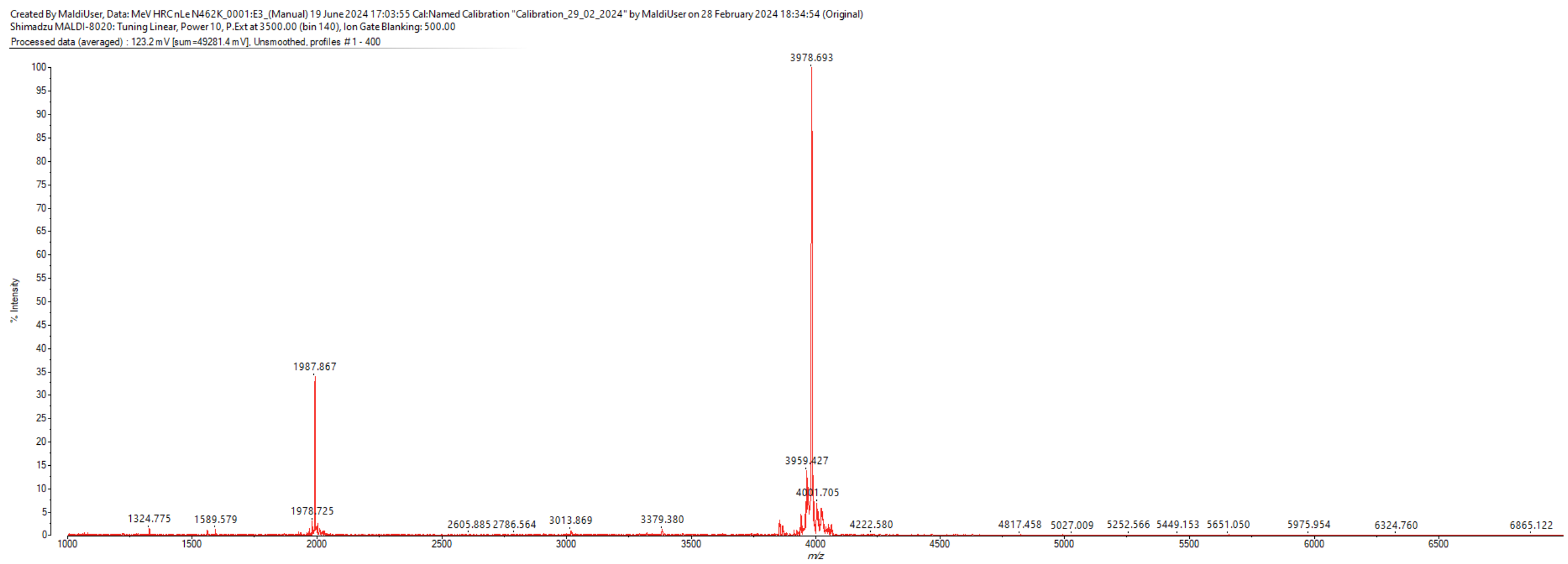

Fig. S21. MALDI-TOF MS spectrum of HR2-N462K.

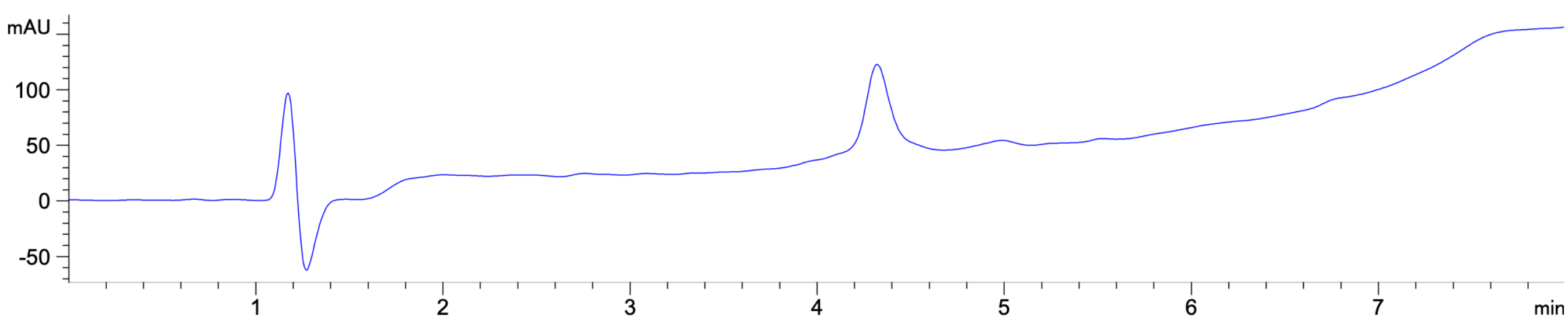

Fig. S22. Analytical HPLC chromatogram of purified HR2-N462K (purity > 95%).

MeV F HR2-N462A

Sequence

Ac-ISLERLDVGTALGNAIAKLEDAKELLESSDQILRS(nLe)-NH_2

Calculated monoisotopic $[M+H]^+$: 3921.1

Calculated monoisotopic $[M+2H]^+$: 1960.6

Observed $[M+2H]^+$: 1961.1

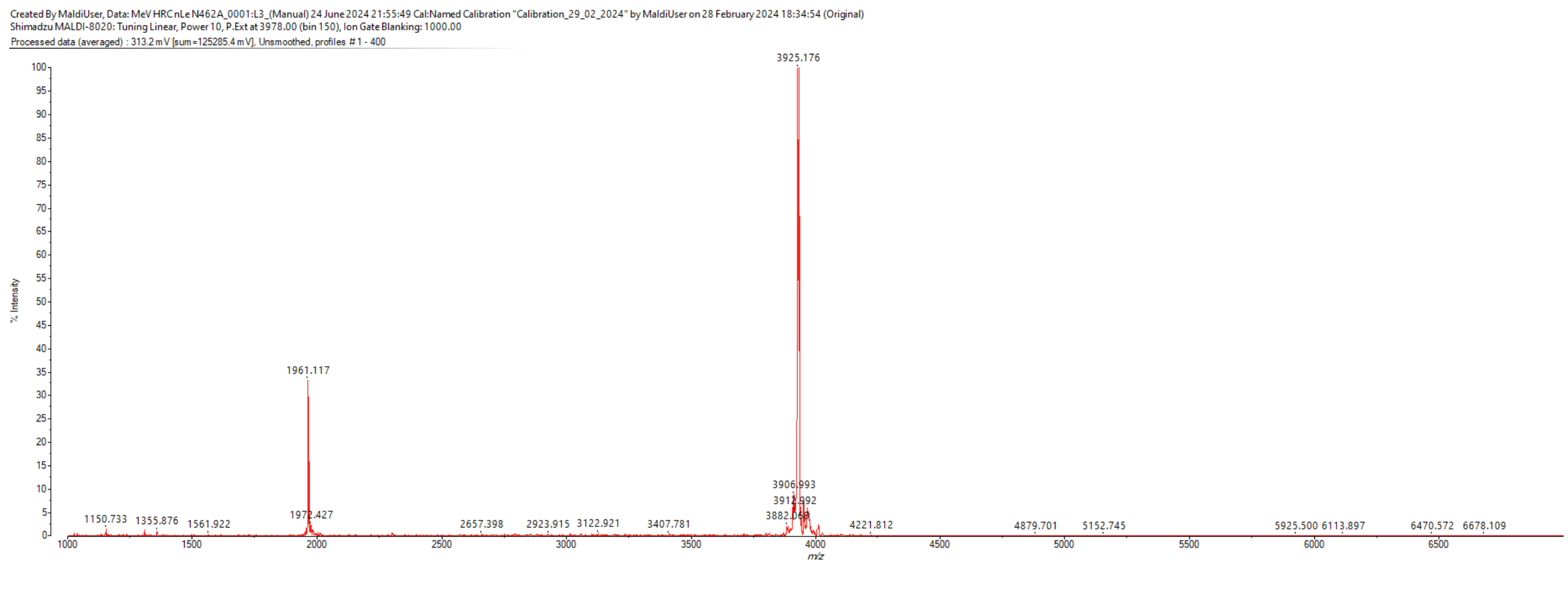

Fig. S23. MALDI-TOF MS spectrum of HR2-N462A.

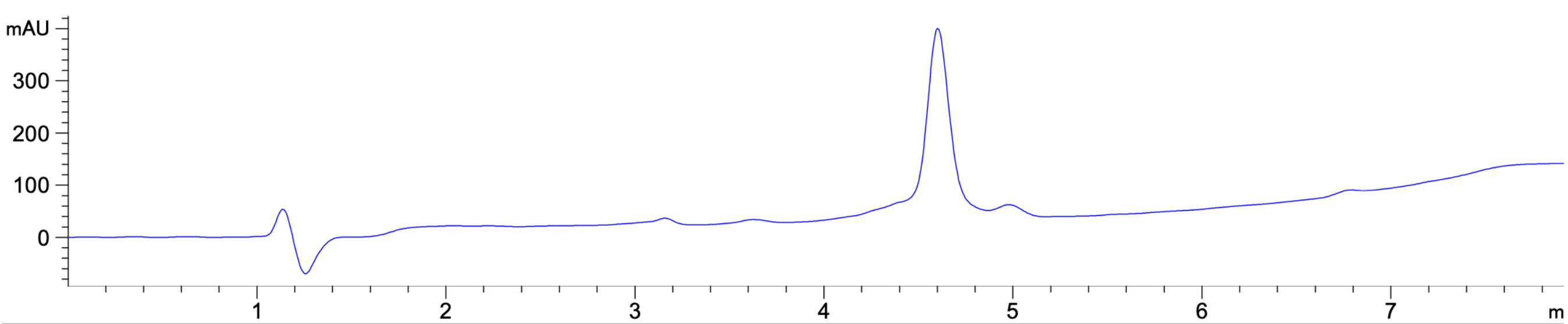

Fig. S24. Analytical HPLC chromatogram of purified HR2-N462A (purity > 95%).

MeV F HR2-N462S

Sequence

Ac-ISLERLDVGTSLGNAIAKLEDAKELLESSDQILRS(nLe)-NH_2

Calculated monoisotopic $[M+H]^+$: 3937.1

Calculated monoisotopic $[M+2H]^+$: 1968.6

Observed $[M+2H]^+$: 1969.4

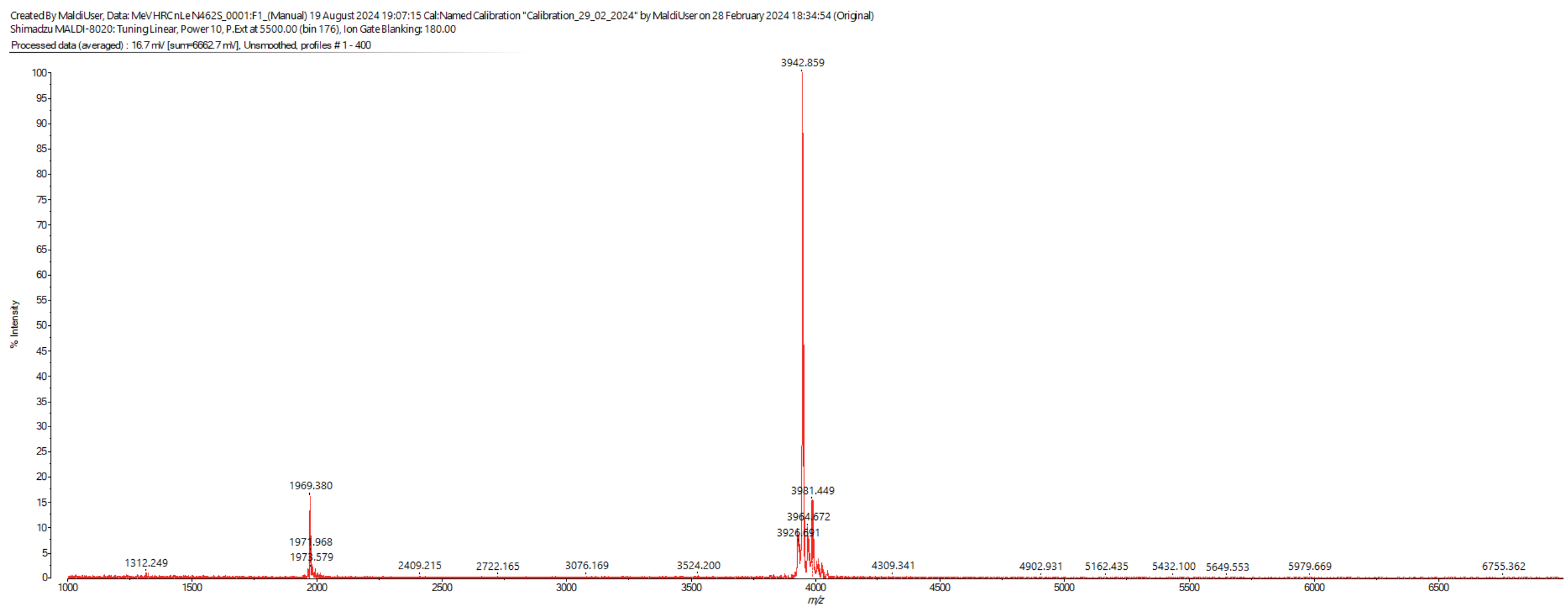

Fig. S25. MALDI-TOF MS spectrum of HR2-N462S.

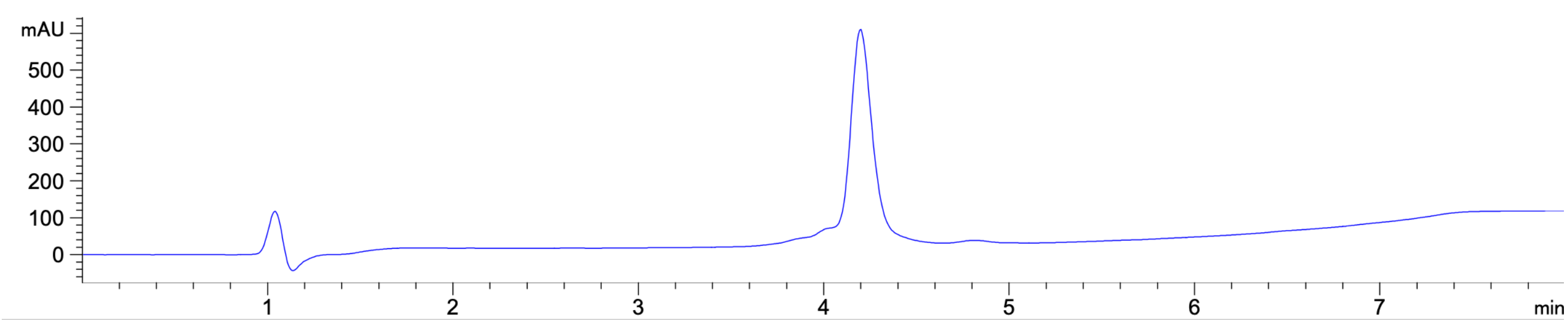

Fig. S26. Analytical HPLC chromatogram of purified HR2-N462S (purity > 95%).

MeV F HR2-N465S

Sequence

Ac-ISLERLDVGTNLGSAIAKLEDAKELLESSDQILRS(nLe)-NH_2

Calculated monoisotopic $[M+H]^+$: 3937.1

Calculated monoisotopic $[M+2H]^+$: 1968.6

Observed $[M+H]^+$: 3939.6

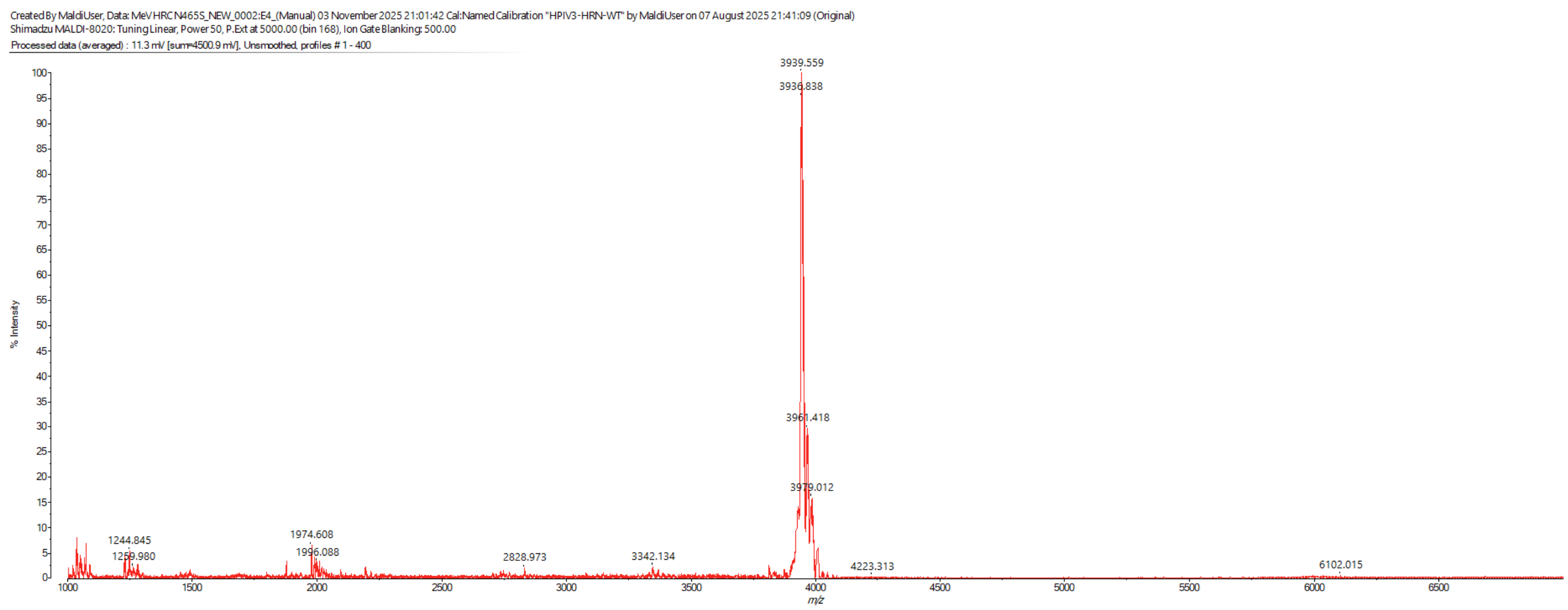

Fig. S27. MALDI-TOF MS spectrum of HR2-N465S.

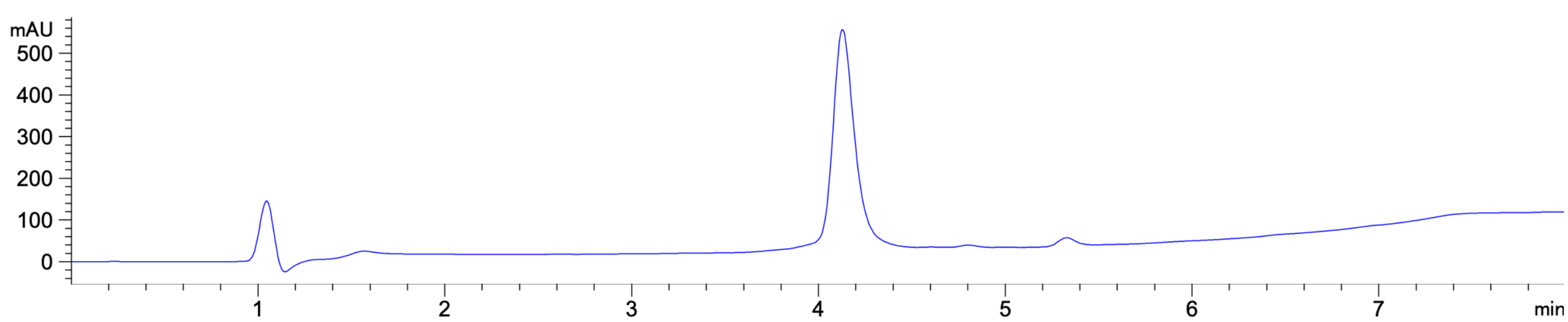

Fig. S28. Analytical HPLC chromatogram of purified HR2-N465S (purity > 95%).

MeV F HR2-T461I/N462S

Sequence

Ac-ISLERLDVG**IS**LGNAIAKLEDAKELLESSDQILRS(nLe)-NH_2

Calculated monoisotopic $[M+H]^+$: 3949.2

Calculated monoisotopic $[M+2H]^+$: 1974.6

Observed $[M+H]^+$: 3951.7

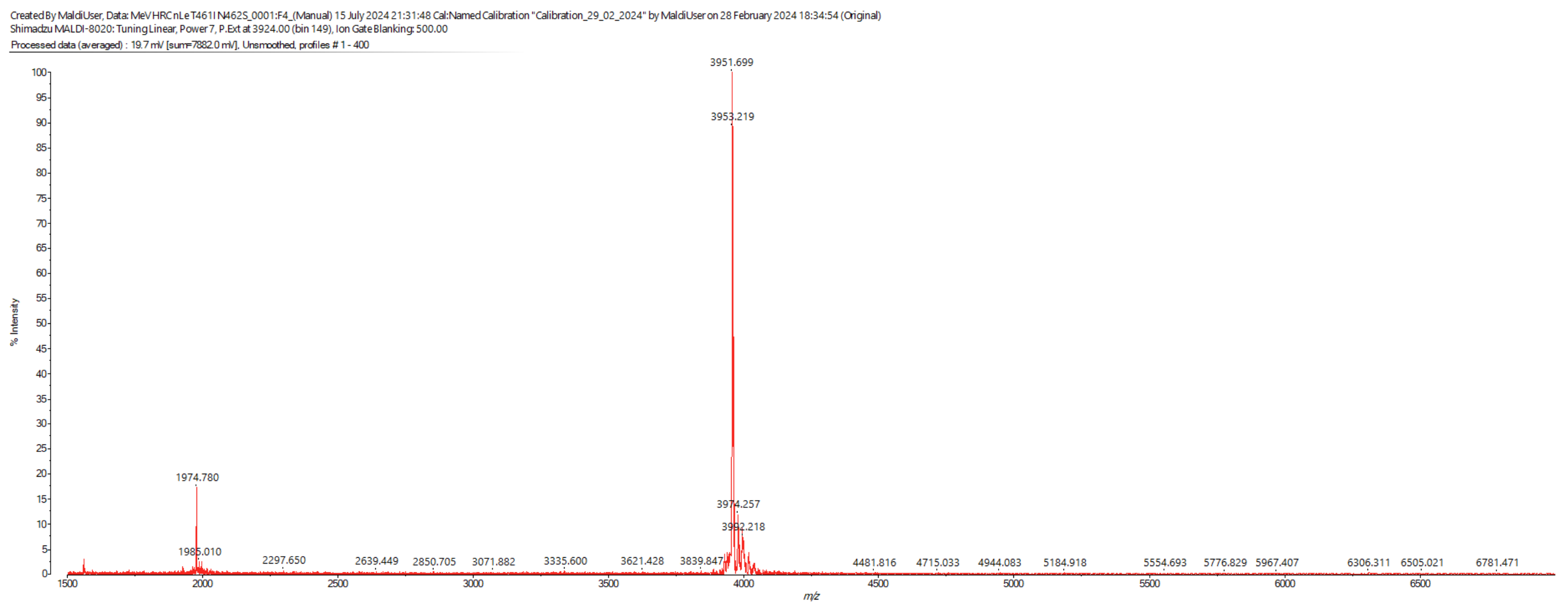

Fig. S29. MALDI-TOF MS spectrum of HR2-T461I/N462S.

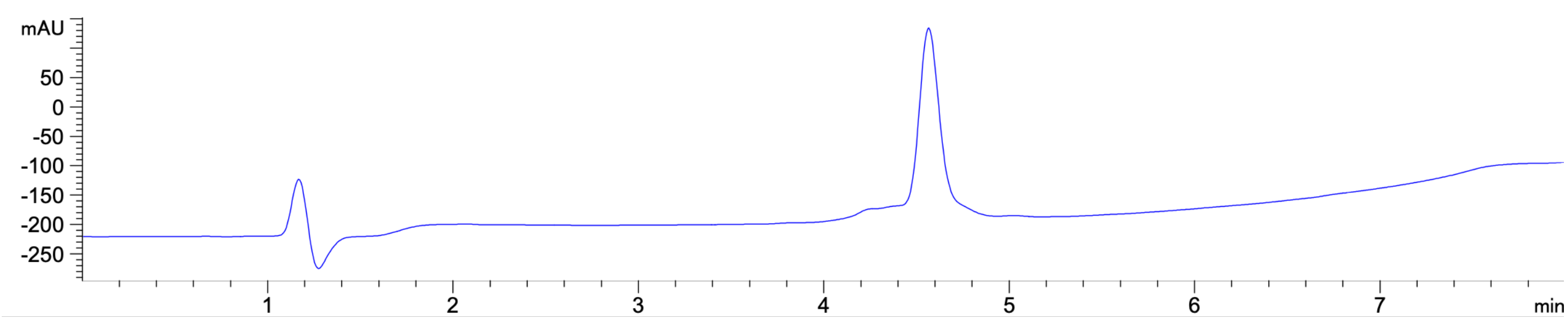

Fig. S30. Analytical HPLC chromatogram of purified HR2-T461I/N462S (purity > 95%).

MeV F HR2-T461I/N465S

Sequence

Ac-ISLERLDVGINLGSAIAKLEDAKELLESSDQILRS(nLe)-NH_2

Calculated monoisotopic $[M+H]^+$: 3949.2

Calculated monoisotopic $[M+2H]^+$: 1974.6

Observed $[M+2H]^+$: 1974.7

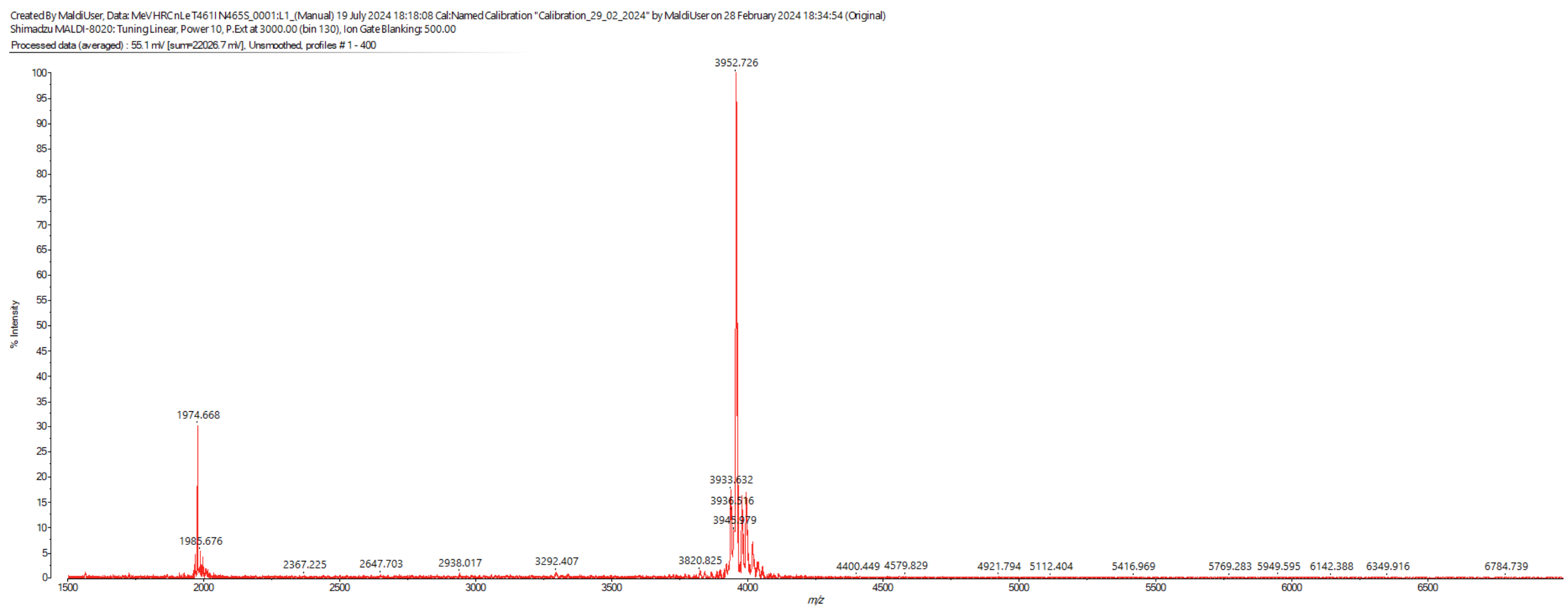

Fig. S31. MALDI-TOF MS spectrum of HR2-T461I/N465S.

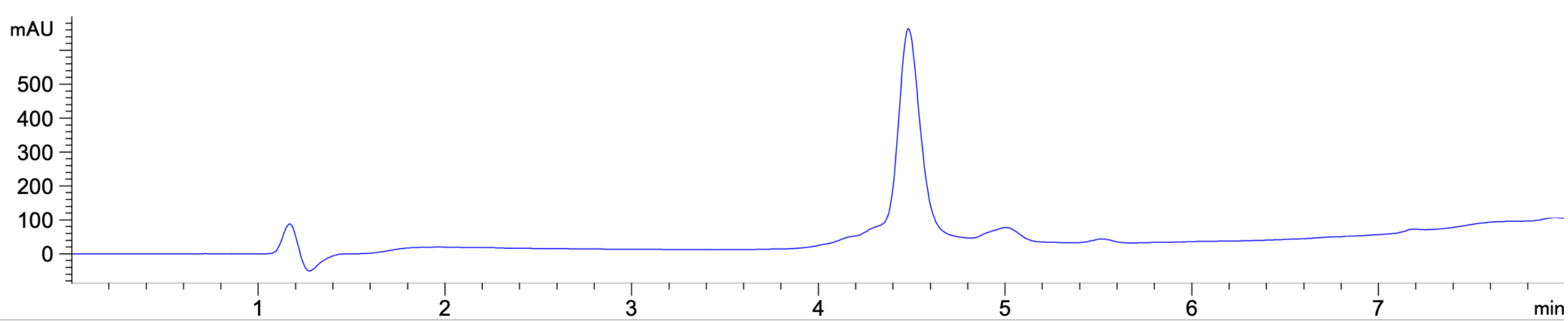

Fig. S32. Analytical HPLC chromatogram of purified HR2-T461I/N465S (purity > 95%).

MeV F HR2-T461I/N462S/N465S

Sequence

Ac-ISLERLDVG**IS**LG**S**AIAKLEDAKELLESSDQILRS(nLe)-NH_2

Calculated monoisotopic $[M+H]^+$: 3924.5

Calculated monoisotopic $[M+2H]^+$: 1962.2

Observed $[M+H]^+$: 3925.9

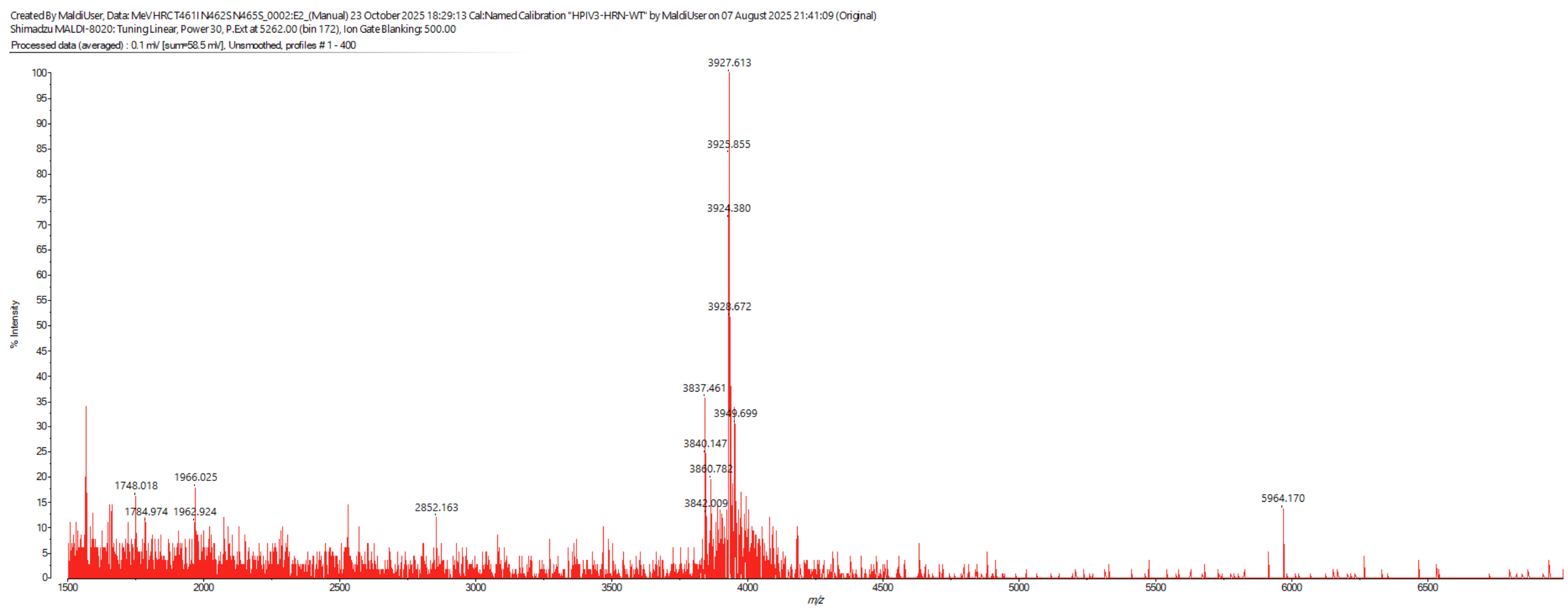

Fig. S33. MALDI-TOF MS spectrum of HR2-T461I/N462S/N465S.

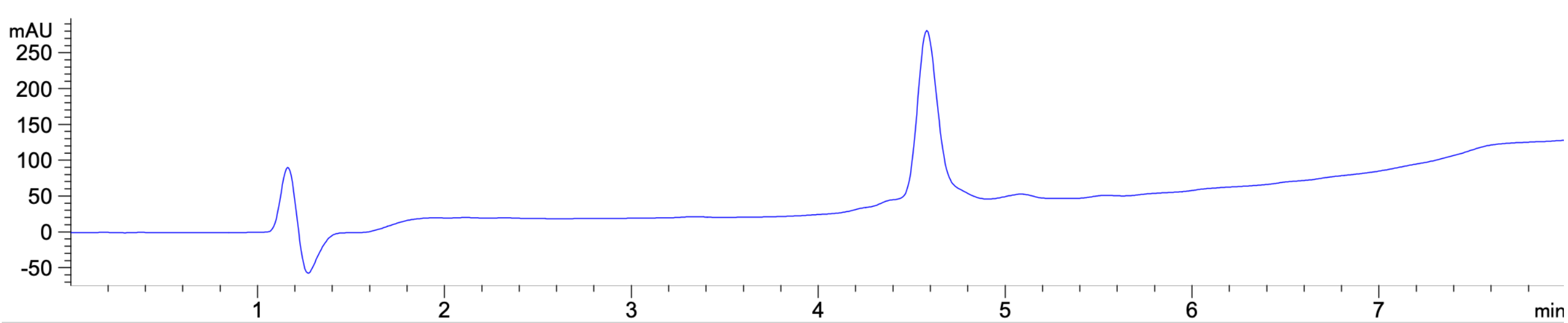

Fig. S34. Analytical HPLC chromatogram of purified HR2-T461I/N462S/N465S (purity > 95%).

MeV F HR2-G464W

Sequence

Ac-ISLERLDVGTNLWNAIAKLEDAKELLESSDQILRS(nLe)-NH_2

Calculated monoisotopic $[M+H]^+$: 4093.2

Calculated monoisotopic $[M+2H]^+$: 2046.6

Observed $[M+H]^+$: 4094.9

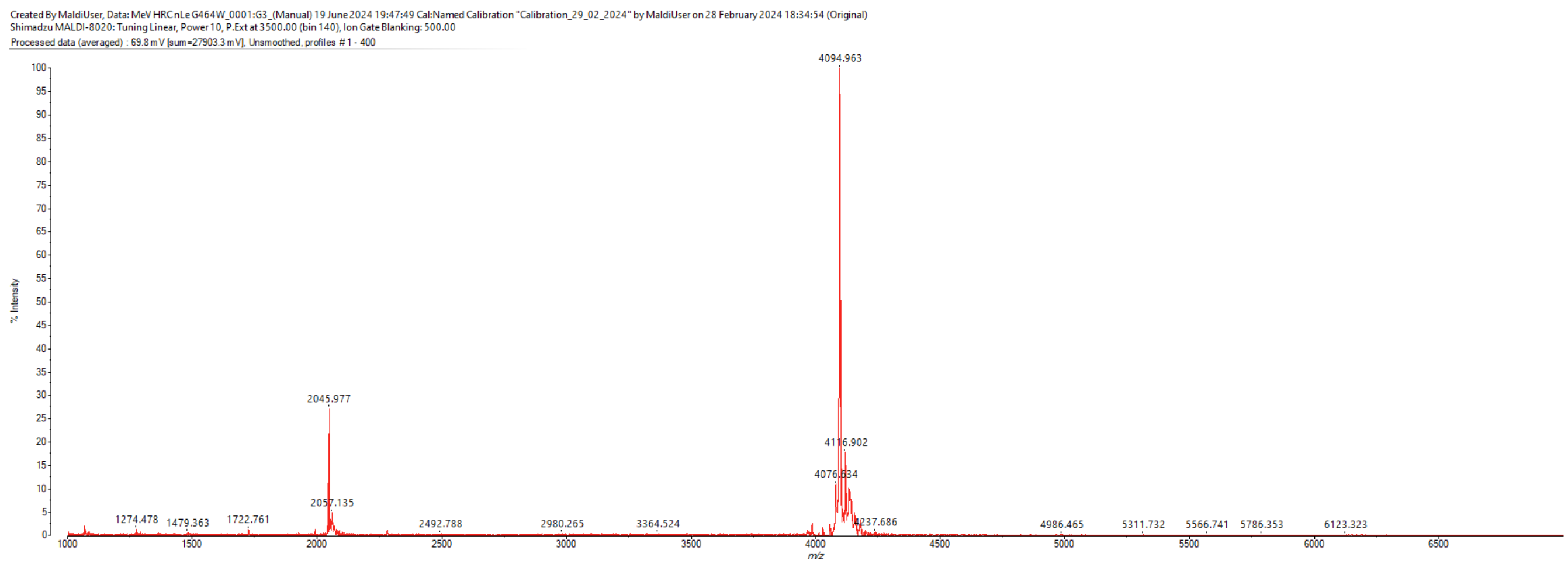

Fig. S35. MALDI-TOF MS spectrum of HR2-G464W.

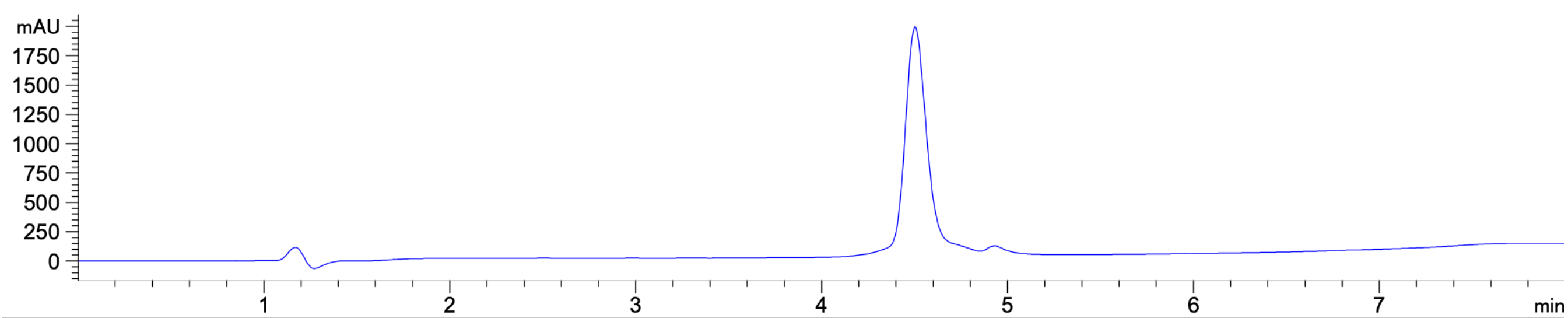

Fig. S36. Analytical HPLC chromatogram of purified HR2-G464W (purity > 95%).

MeV F HR2-G464A

Sequence

Ac-ISLERLDVGTNLANAIAKLEDAKELLESSDQILRS(nLe)-NH_2

Calculated monoisotopic $[M+H]^+$: 3978.2

Calculated monoisotopic $[M+2H]^+$: 1989.1

Observed $[M+H]^+$: 3978.7

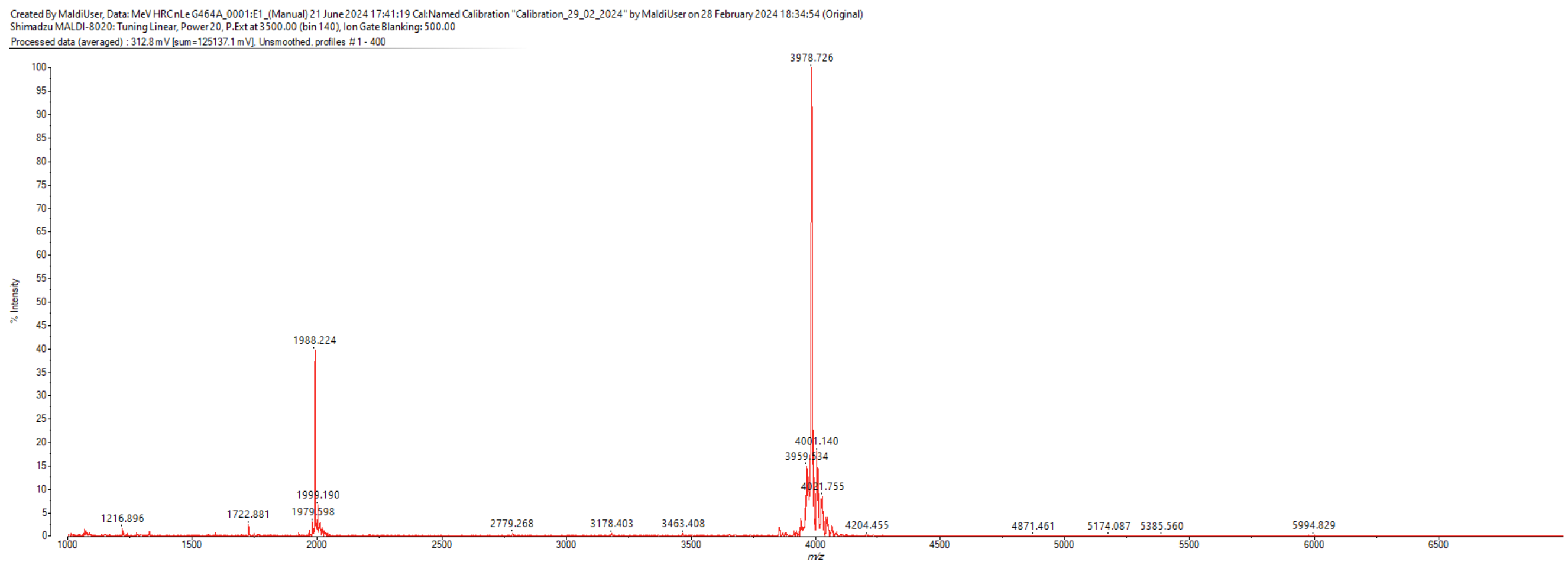

Fig. S37. MALDI-TOF MS spectrum of HR2-G464A.

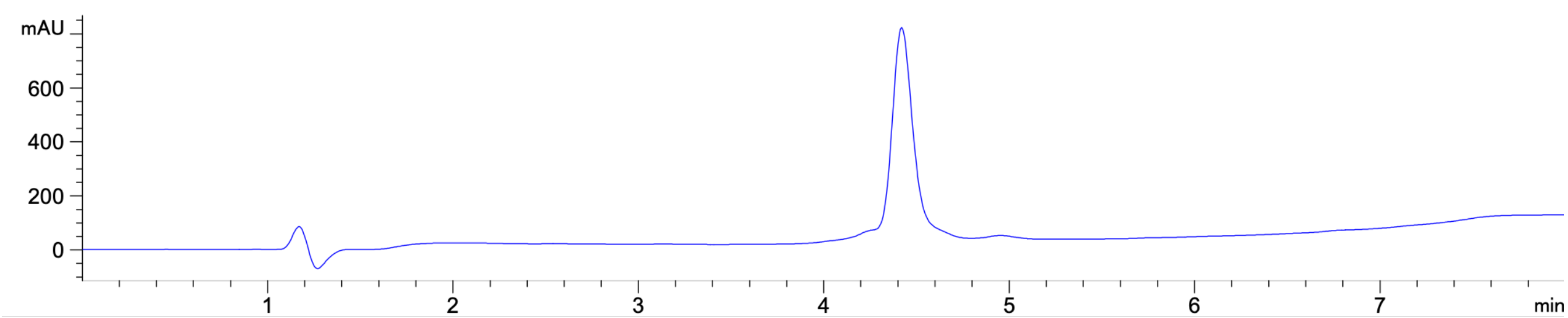

Fig. S38. Analytical HPLC chromatogram of purified HR2-G464A (purity > 95%).

Circular Dichroism Experiments

Instrumentation

All circular dichroism (CD) spectroscopy experiments were performed on a JASCO J-1500 CD spectrophotometer using 1 mm high performance quartz glass cuvettes (Hellma) with a spectral range of 200–2500 nm and a chamber volume of 400 µL.

Wavelength Scan

CD samples were prepared using 1:1 mixtures of MeV HR1-wt (0.5-1 µg/µL) and HR2 peptide variants (0.5-1 µg/µL) at 25 µM concentrations in PBS (pH 7.0) with 2.5% hexafluoro-2-propanol (HFIP). Wavelength scans were collected at 25 °C from 250 nm to 200 nm with a 1 nm bandwidth. Experiments were performed in triplicate with individual spectra shown in Figure S39.

Thermal Denaturation

CD samples were prepared using 1:1 mixture of MeV HR1-wt (0.5-1 µg/µL) and HR2 peptide variants (0.5-1 µg/µL) at 25 µM concentrations in PBS (pH 7.0) with 2.5% hexafluoro-2-propanol (HFIP). Variable-temperature circular dichroism (vt-CD) spectra for each co-assembly were performed by monitoring ellipticity at 222 nm as a function of temperature from 10–90 °C in 2 °C increments with a 1.00 °C/min rate, 20-second equilibration time, and a 0.15 °C tolerance. All experiments were performed in triplicates. Data were fit to a two-state folding model using GraphPad Prism 10 and apparent melting temperature ($T_{m,app}$) values, defined as the mid-point of each transition, were determined. Experiments were performed in triplicate with individual spectra shown in Figure S40.

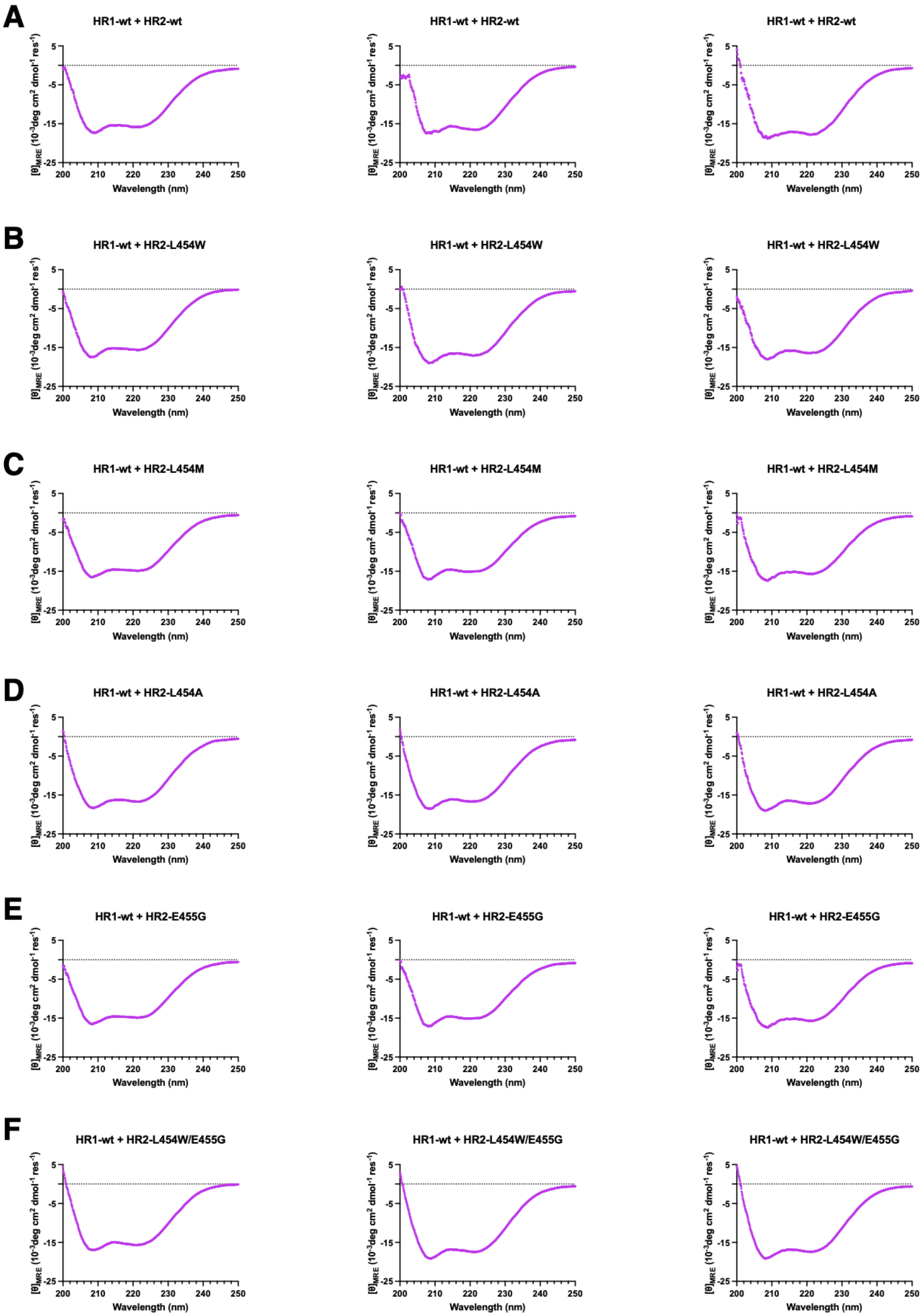
A
HR1-wt + HR2-wt
HR1-wt + HR2-wt
HR1-wt + HR2-wt
B
HR1-wt + HR2-L454W
HR1-wt + HR2-L454W
HR1-wt + HR2-L454W
C
HR1-wt + HR2-L454M
HR1-wt + HR2-L454M
HR1-wt + HR2-L454M
D
HR1-wt + HR2-L454A
HR1-wt + HR2-L454A
HR1-wt + HR2-L454A
E
HR1-wt + HR2-E455G
HR1-wt + HR2-E455G
HR1-wt + HR2-E455G
F
HR1-wt + HR2-L454W/E455G
HR1-wt + HR2-L454W/E455G
HR1-wt + HR2-L454W/E455G
[θ]MRE (10-3deg cm2 dmol-1 res-1)
Wavelength (nm)
5
-5
-15
-25
200
210
220
230
240
250

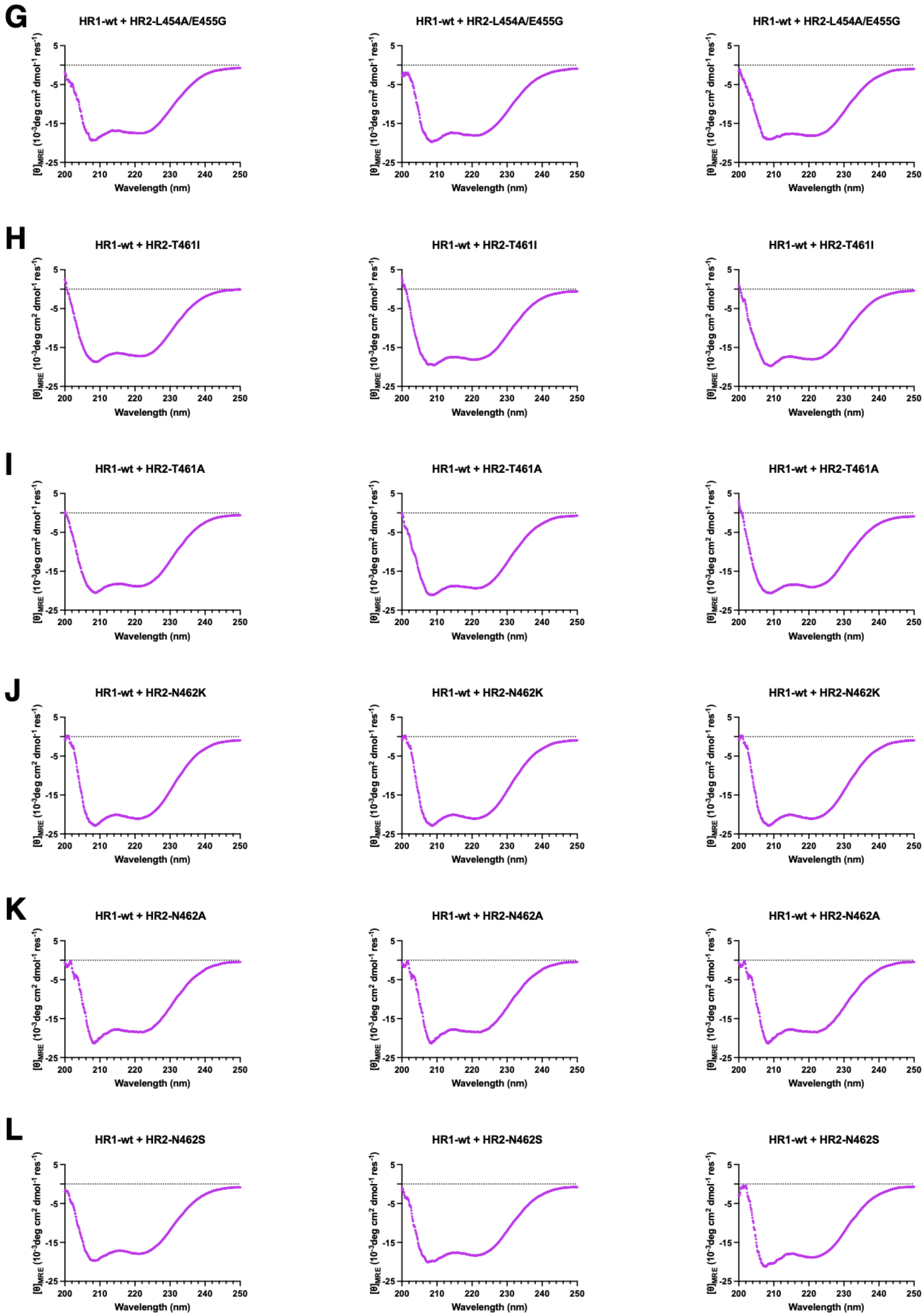
G
HR1-wt + HR2-L454A/E455G
H
HR1-wt + HR2-T461I
I
HR1-wt + HR2-T461A
J
HR1-wt + HR2-N462K
K
HR1-wt + HR2-N462A
L
HR1-wt + HR2-N462S
[θ]MRE (10-3deg cm2 dmol-1 res-1)
Wavelength (nm)
5
-5
-15
-25
200
210
220
230
240
250

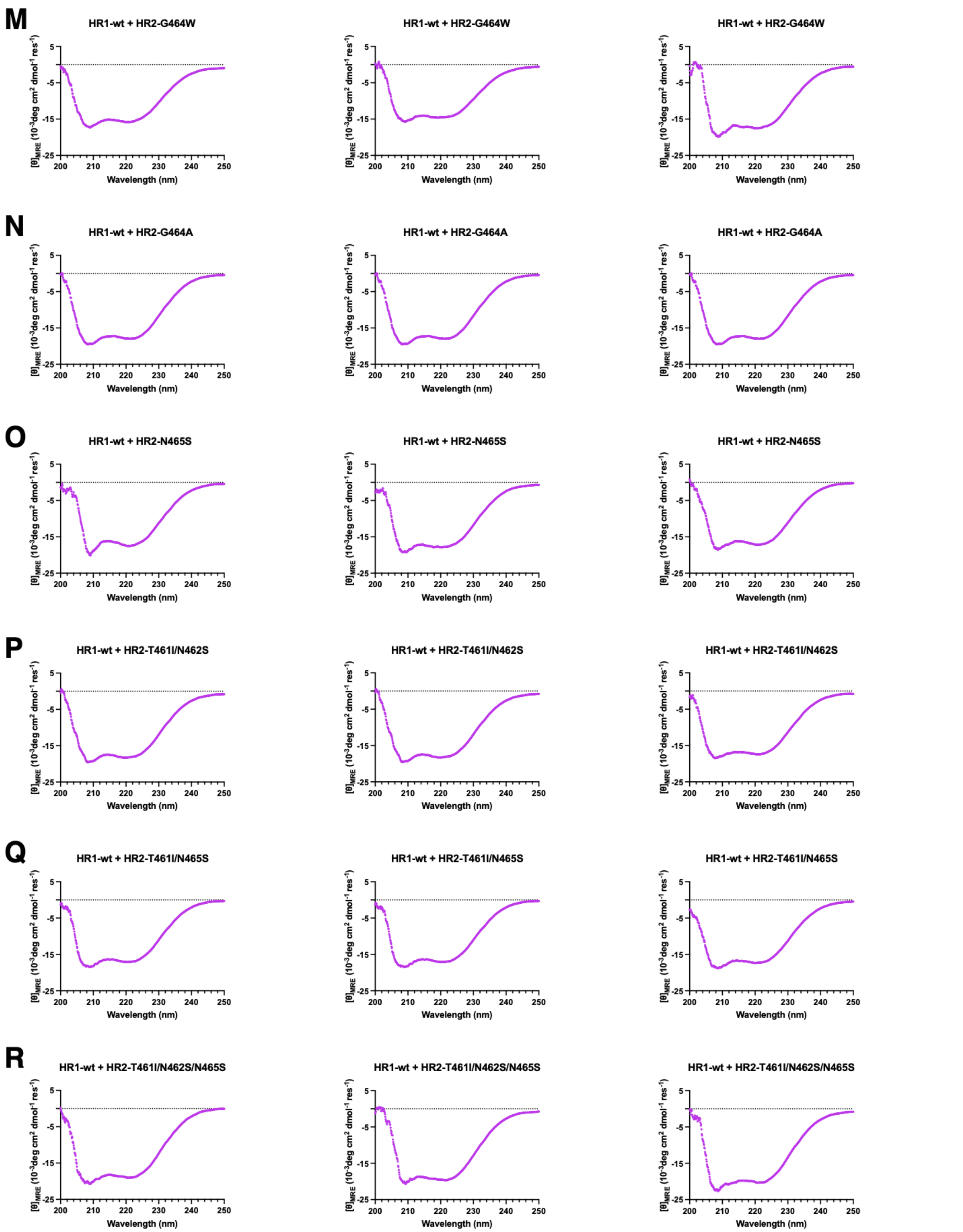

Figure S39. CD wavelength scans of 1:1 mixtures of HR1-wt with HR2 variants at 25 μM in PBS (pH 7.0) + 2.5% HFIP. All experiments were performed in triplicate.

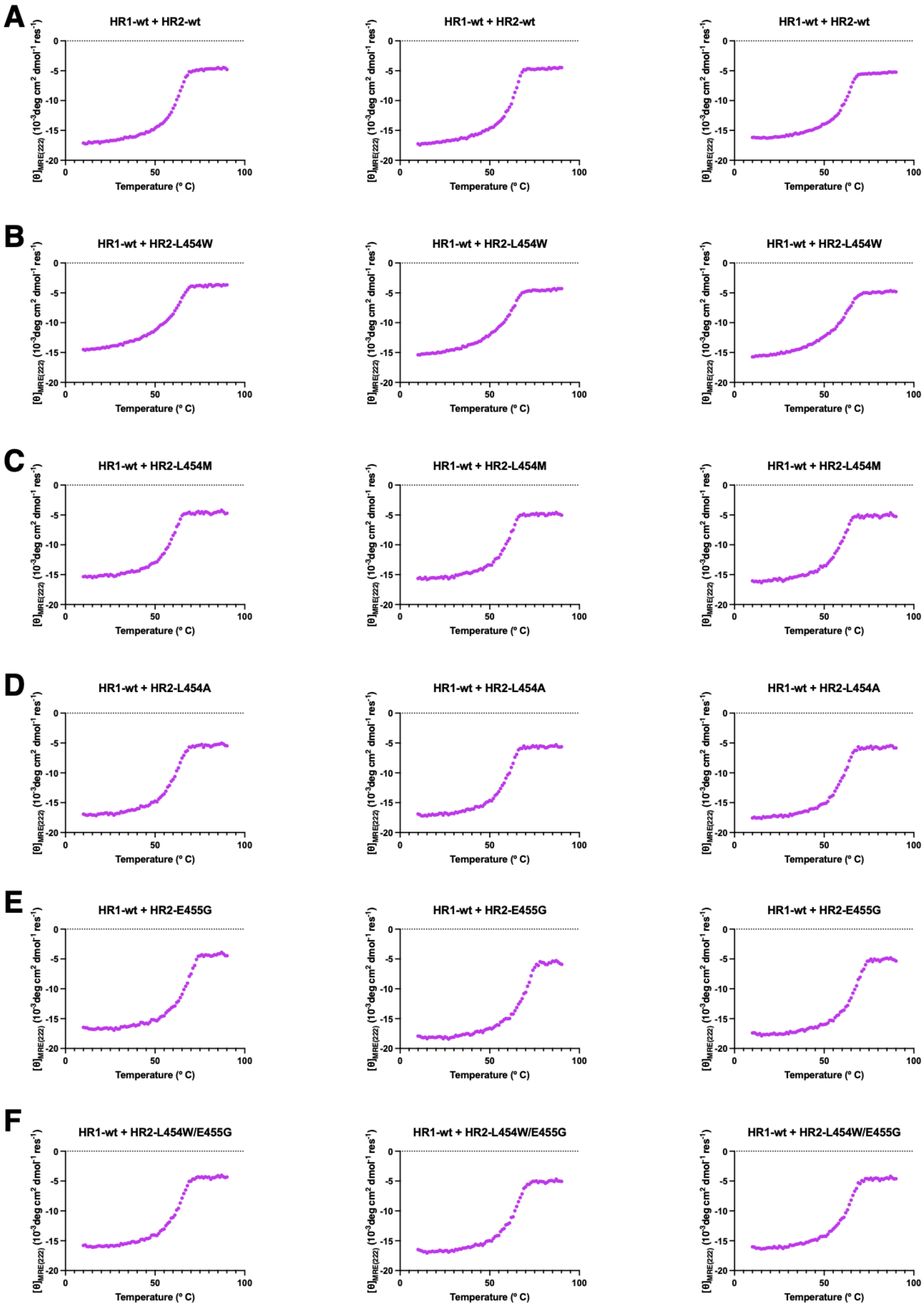
A
HR1-wt + HR2-wt
HR1-wt + HR2-wt
HR1-wt + HR2-wt
B
HR1-wt + HR2-L454W
HR1-wt + HR2-L454W
HR1-wt + HR2-L454W
C
HR1-wt + HR2-L454M
HR1-wt + HR2-L454M
HR1-wt + HR2-L454M
D
HR1-wt + HR2-L454A
HR1-wt + HR2-L454A
HR1-wt + HR2-L454A
E
HR1-wt + HR2-E455G
HR1-wt + HR2-E455G
HR1-wt + HR2-E455G
F
HR1-wt + HR2-L454W/E455G
HR1-wt + HR2-L454W/E455G
HR1-wt + HR2-L454W/E455G
[θ]MRE(222) (10-3deg cm2 dmol-1 res-1)
Temperature (° C)
0
-5
-10
-15
-20
0
50
100

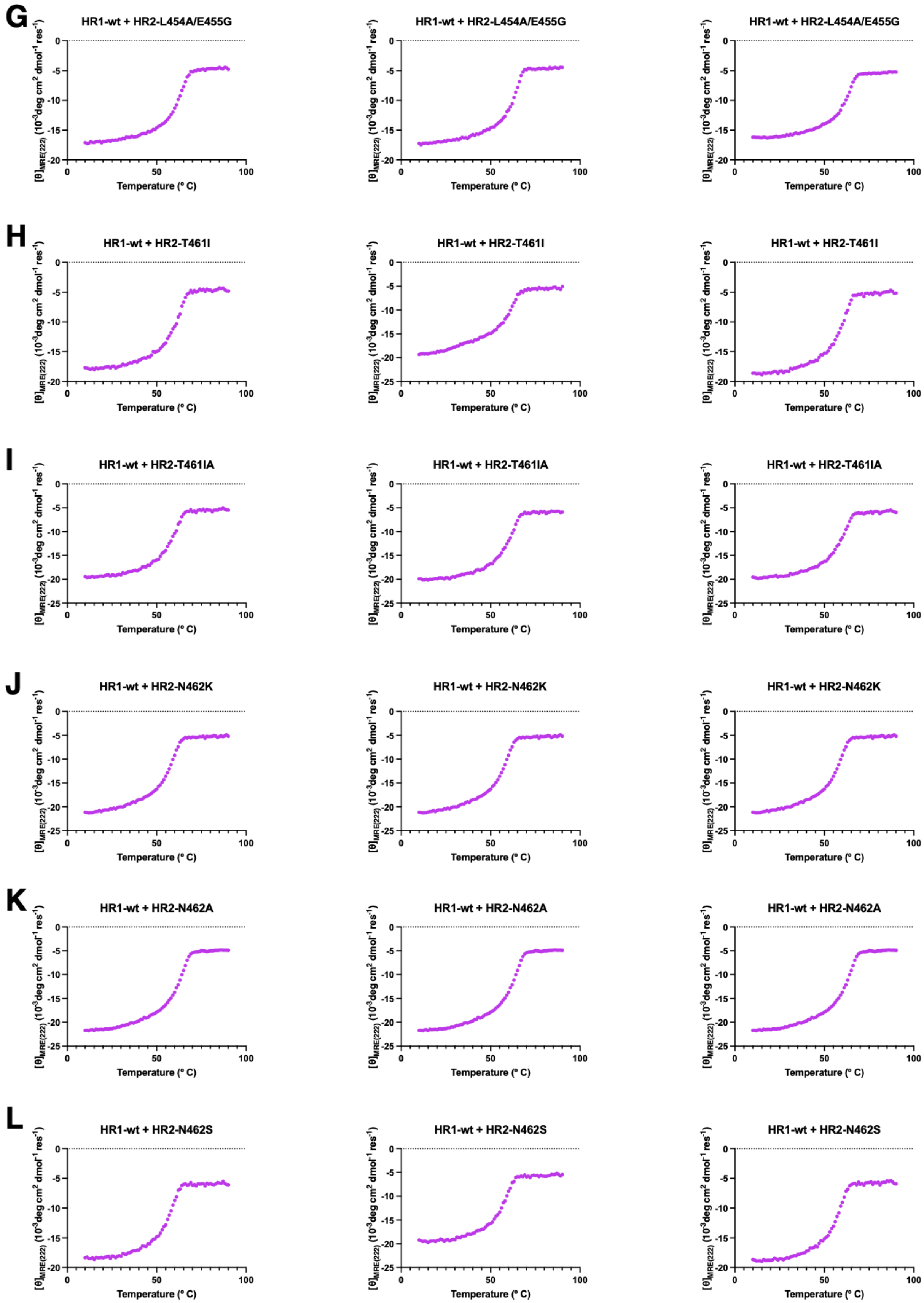
G
HR1-wt + HR2-L454A/E455G
HR1-wt + HR2-L454A/E455G
HR1-wt + HR2-L454A/E455G
H
HR1-wt + HR2-T461I
HR1-wt + HR2-T461I
HR1-wt + HR2-T461I
I
HR1-wt + HR2-T461IA
HR1-wt + HR2-T461IA
HR1-wt + HR2-T461IA
J
HR1-wt + HR2-N462K
HR1-wt + HR2-N462K
HR1-wt + HR2-N462K
K
HR1-wt + HR2-N462A
HR1-wt + HR2-N462A
HR1-wt + HR2-N462A
L
HR1-wt + HR2-N462S
HR1-wt + HR2-N462S
HR1-wt + HR2-N462S
$[\theta]_{MRE(222)}$ (10^{-3}deg cm^2 dmol^{-1} res^{-1})
Temperature (° C)

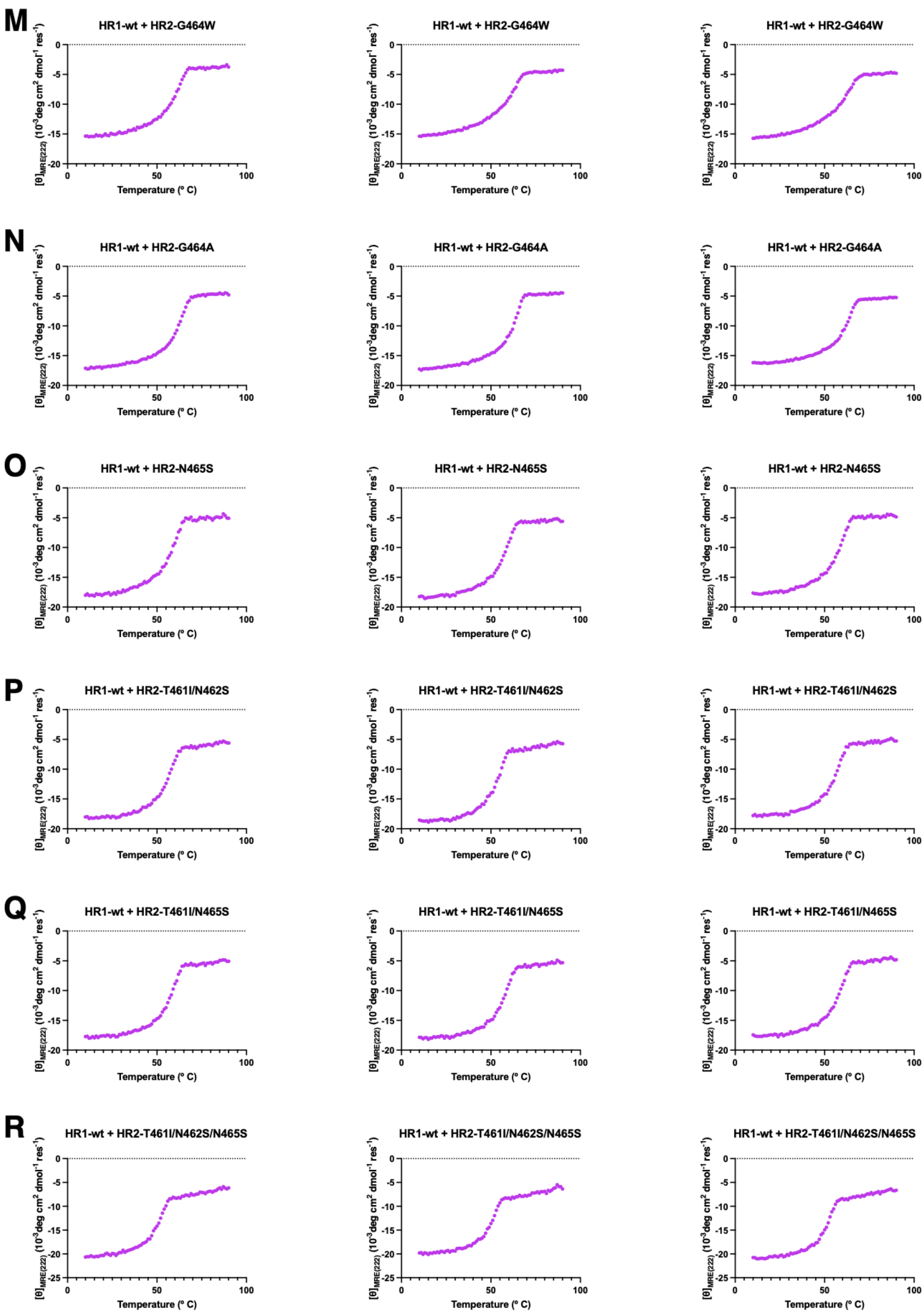

Figure S40. Temperature-dependent denaturation of 1:1 mixtures of HR1-wt with HR2 variants at 25 μM in PBS (pH 7.0) + 2.5% HFIP. All experiments were performed in triplicate.

Table S1. Apparent Melting Temperature of MeV Fusion Core Assemblies

MeV Variant[a]	$T_{m,app}$ (°C)[b]	Δ $T_{m,app}$ (°C)[c]	Adjusted p-value	Significant
Wild type	60.6(±0.3)	-	-	-
L454W	57.2(±0.5)	-3.4	<0.0001	Yes
L454M	57.6(±0.4)	-3.0	<0.0001	Yes
E455G	65.1(±0.2)	+4.5	<0.0001	Yes
L454W/E455G	61.5(±0.5)	+0.9	0.0258	Yes
T461A	57.7(±0.4)	-2.9	<0.0001	Yes
T461I	60.1(±0.4)	-0.5	0.4781	No
N462K	55.0(±0.2)	-5.6	<0.0001	Yes
N462S	55.3(±0.2)	-5.3	<0.0001	Yes
N465S	56.1(±0.2)	-4.5	<0.0001	Yes
T461I/N462S	54.9(±0.2)	-5.7	<0.0001	Yes
T461I/N465S	56.4(±0.3)	-4.2	<0.0001	Yes
T461I/N462S/N465S	50.3(±0.5)	-10.3	<0.0001	Yes
G464W	57.7(±0.3)	-2.9	<0.0001	Yes

[a]Coiled-coil assemblies were created using 1:1 mixtures of MeV HR1-wt and MeV HR2 peptide variants. Variants listed indicate mutations within the MeV HR2 peptide. [b]Variable-temperature circular dichroism (vt-CD) experiments were performed by monitoring ellipticity at 222 nm for each co-assembly as a function of temperature. Peptide concentrations are 25 µM in PBS (pH 7.0) + 2.5% HFIP (v/v). $T_{m,app}$ values are reported as the mean ± SD from three independent experiments. Statistical comparisons among variants were performed using of one-way ANOVA followed by Dunnett's multiple-comparison test with the wild type assembly as the control. ($p<0.05$). [c]$\Delta T_{m,app}$ values were calculated relative to the wild-type value [$\Delta T_{m,app} = T_{m,app}$(wild type) – $T_{m,app}$(variant)]. Negative $\Delta T_{m,app}$ values indicate destabilization.

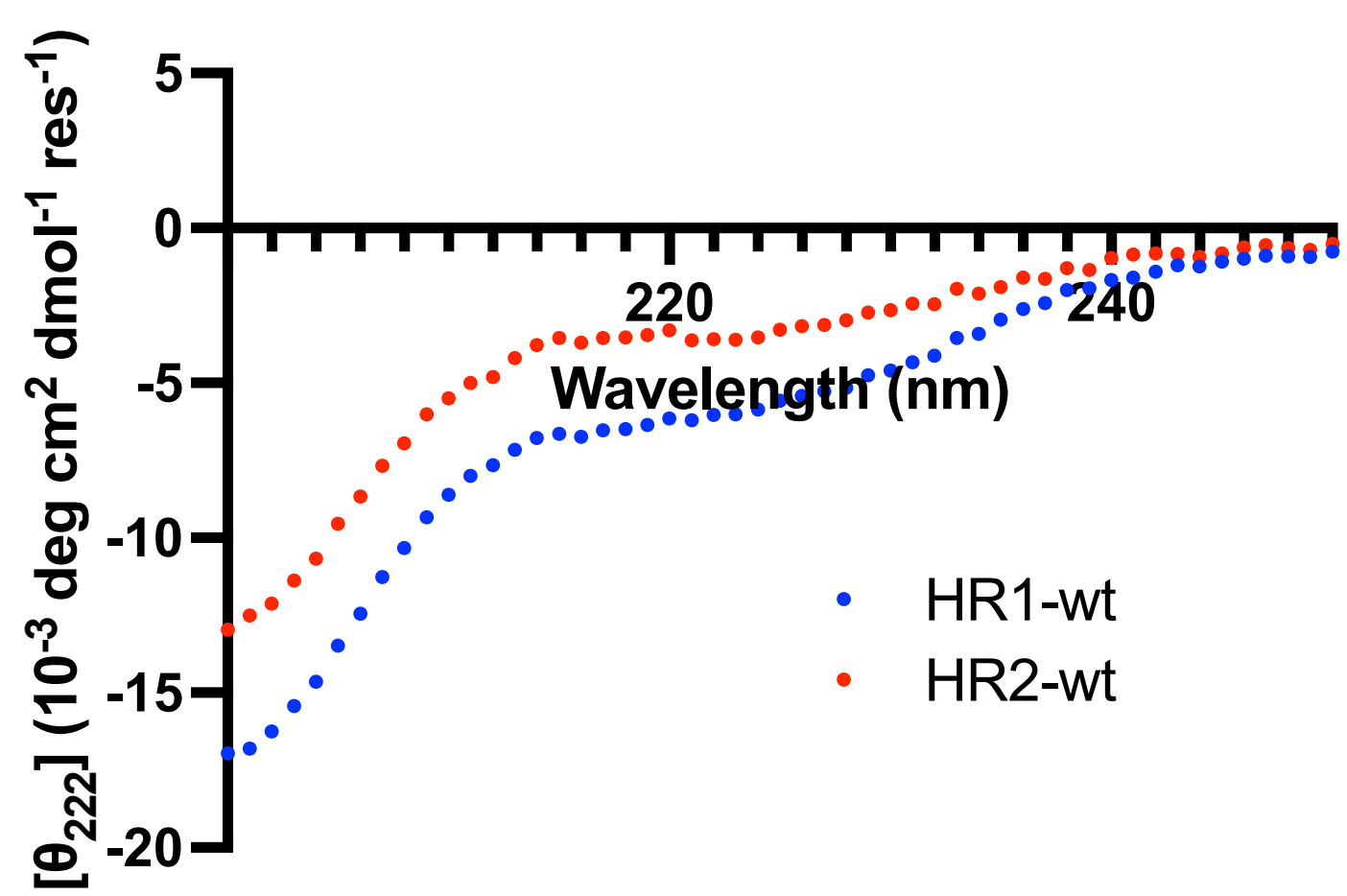

Fig.S41. Representative circular dichroism studies of MeV HR1-wt and HR2-wt individual peptides. Wavelength scans of HR1-wt (blue) and HR2-wt (green) at 25 µM in PBS (pH 7.0) demonstrate non-helical/ random coil structure.

X-ray Crystallography

Co-crystallization

A co-crystallization solution was prepared containing a 1:1 mixture of MeV HR1-wt and HR2 peptide variants in water at a total concentration of 5-12 mg/mL. Crystals were grown by hanging drop vapor diffusion using a crystallization condition optimized from Molecular Dimensions Morpheus crystallization screen. A 2 µL drop comprising a 1:1 mixture of co-crystallization solution and the crystallization condition was placed on a glass cover slide that was then inverted to seal a well containing 150 µL of the crystallization condition. Crystallization conditions for each structure are provided in **Table S2**.

Table S2. Crystallization Conditions

Variant	Additives	Buffer System	Precipitant
Wild-type	0.09M Sodium fluoride; 0.09M Sodium bromide; 0.09M Sodium iodide	0.1M Imidazole; MES monohydrate (acid), pH 6.5	37.5% v/v MPD; 37.5% PEG 1000; 37.5% w/v PEG 3350
L454W	0.06M Magnesium chloride hexahydrate; 0.06M Calcium chloride dihydrate	0.1M Imidazole; MES monohydrate (acid), pH 6.5	37.5% v/v MPD; 37.5% PEG 1000; 37.5% w/v PEG 3350
T461I	0.12M 1,6-Hexanediol; 0.12M 1-Butanol; 0.12M 1,2-Propanediol; 0.12M 2-Propanol; 0.12M 1,4-Butanediol; 0.12M 1,3-Propanediol	0.1M Imidazole; MES monohydrate (acid), pH 6.5	30% v/v, PEG 500 MME; 30% w/v PEG 20000
T461A	0.09M Sodium fluoride; 0.09M Sodium bromide; 0.09M Sodium iodide	0.1M Imidazole; MES monohydrate (acid), pH 6.5	37.5% v/v MPD; 37.5% PEG 1000; 37.5% w/v PEG 3350
N462K	0.09M Sodium fluoride; 0.09M Sodium bromide; 0.09M Sodium iodide	0.1M Tris (base); BICINE, pH 8.5	30% v/v, PEG 500 MME; 30% w/v PEG 20000
G464W	0.12M 1,6-Hexanediol; 0.12M 1-Butanol; 0.12M 1,2-Propanediol; 0.12M 2-Propanol; 0.12M 1,4-Butanediol; 0.12M 1,3-Propanediol	0.1M Imidazole; MES monohydrate (acid), pH 6.5	37.5% v/v MPD; 37.5% PEG 1000; 37.5% w/v PEG 3350
T461I/N462S/N465S	0.06M Magnesium chloride hexahydrate; 0.06M Calcium chloride dihydrate	0.1M Sodium HEPES; MOPS (acid), pH 7.5	30% v/v, PEG 500 MME; 30% w/v PEG 20000

Crystal Morphologies

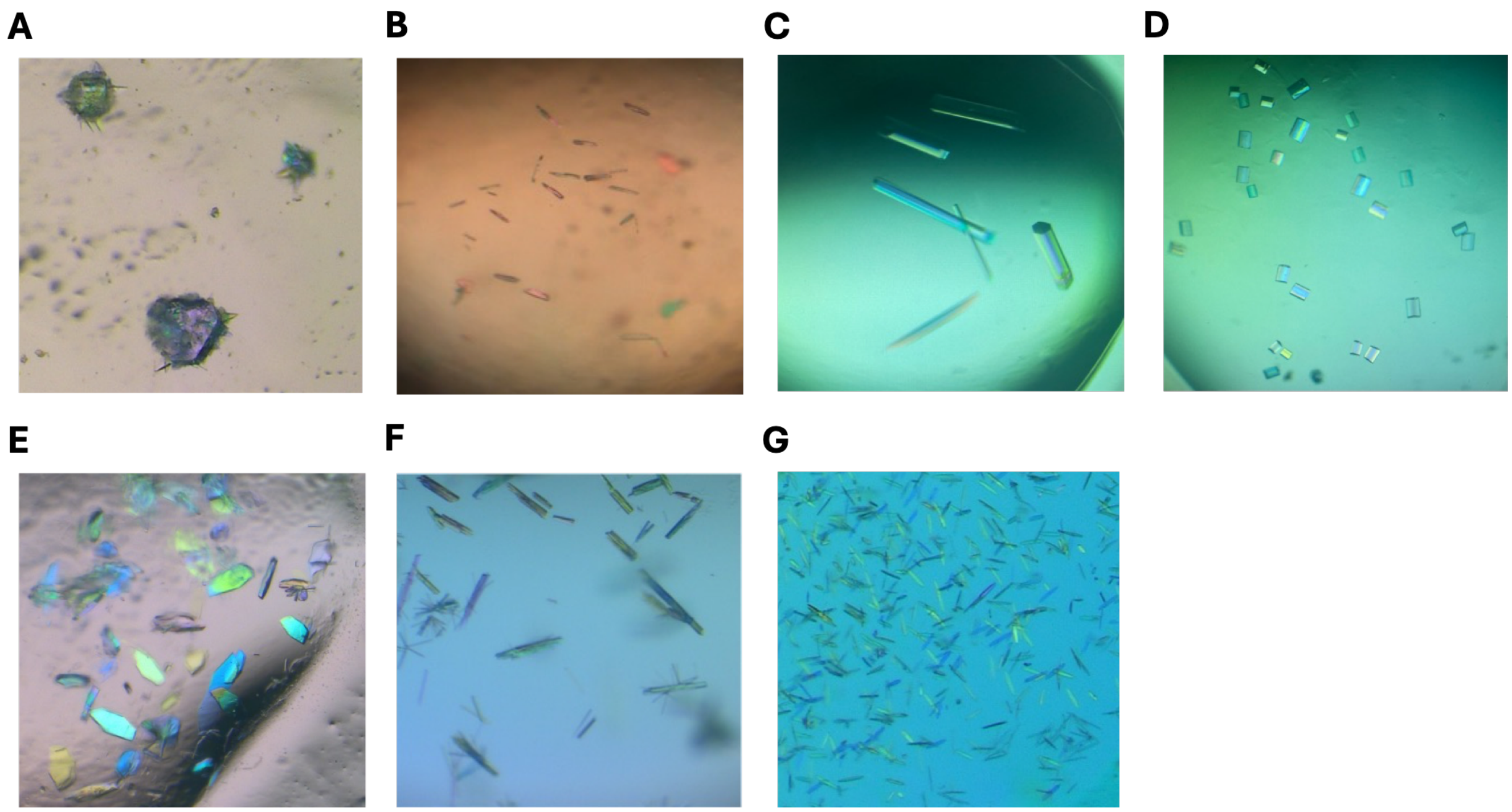

Fig. S42. Crystal morphology of MeV postfusion core variants (A) wild-type (B) L454W (C) N462K (D)T461I (E) T461A (F) T461I/N462S/N465S (G) G464W.

X-ray Data Collection, Processing, Structure Solution, and Refinement

Crystals were looped and vitrified in liquid nitrogen. Diffraction data was collected at the National Synchrotron Light Source II (NSLS-II) at Brookhaven National Laboratory (BNL) on the AMX beam line (17-ID-1). Data were indexed and integrated using the program *XDS*.[1] Data were scaled and merged using the program *AutoProc*.[2] The molecular replacement solutions were found with the program *Phaser* using a poly-alanine dimers constructed from PDB entries given in **Table S3** as search models. Model refinement was carried out using the program *phenix.refine*[3] in combination with manual real-space model building and refinement in the program *Coot*.[4]

Table S3. Search Models for Molecular Replacement

Crystal Structure		Search Model	
**PDB ID**	**Variant**	**PDB ID**	**Structure**
9ZUH	L454W	6NRO	Human parainfluenza virus type 3 fusion protein N-terminal heptad repeat domain + VIQKI
9YD9	T461I	9ZUH	Measles virus fusion glycoprotein (L454W variant)
9Q4F	Wild-type	9YD9	Measles virus fusion glycoprotein (T461I variant)
9YG6	T461A	9YD9	Measles virus fusion glycoprotein (T461I variant)
9YFP	N462K	8X03	Crystal structure of measles virus fusion inhibitor M1 complexed with F protein HR1 (HR1-47) (P321 space group)
9Y9O	G464W	9ZUH	Measles virus fusion glycoprotein (L454W variant)
9YAC	T461I/N462S/N465S	9ZUH	Measles virus fusion glycoprotein (L454W variant)

Table S4. Search Models for Molecular Replacement

Variant	Backbone RMSD vs WT (Å)	Solvent Accessible Area (Å^2)[a]	Buried Surface Area (Å^2)[a]
WT	-	10840	12830
L454W	0.241	10500	12670
T461A	0.226	10750	12680
T461I	0.246	11162	12341
N462K	0.282	10560	12370
T461I/N462S/N465S	0.273	11240	12520
G464W	0.280	11540	12520

[a] Solvent accessible and buried interface area calculations were performed for each A_3B_3 hexameric assembly using PDBePISA.[5]

Table S5. Measles Virus Fusion Glycoprotein (Wild-type variant): PDB 9Q4F

**Data collection**	
X-ray source	NSLS-II 17-ID-1
X-ray detector	Dectris Eiger X 9M
Detector distance (mm)	190.05
Oscillation range (º)	0.1
Wavelength (Å)	0.91969
Space group	P 1 21 1
a / b / c (Å)	49.6 / 54.0 / 71.0
α / β / γ (º)	90.0 / 92.7 / 90.0
Resolution range (Å)	32.91–2.11 (2.14–2.11)
Number of observations	95363 (4418)
Unique reflections	21754 (1087)
Completeness	99.7 (99.5)
Redundancy	4.4 (4.1)
Mean $I/\sigma(I)$	5.9 (1.2)
CC1/2*	0.997 (0.975)
R_{merge}	0.14 (1.0)
R_{meas}	0.18 (1.32)
R_{pim}	0.12 (0.85)
**Refinement statistics**	
Refinement program	Phenix.refine: 1.20.1-4487-000
Resolution range (Å)	32.91–2.11
Data cutoff (sigma (F))	1.35
Completeness (working+test) (%)	77.92
Number of unique reflections refined against	21415
R-value (work)	0.1959
R-value (free)	0.2514
R-value (work + test)	0.2012
Free R value test set count	2000
Mean B value (overall $Å^2$)	34.75

Table S6. Measles Virus Fusion Glycoprotein (T461I variant): PDB 9YD9

**Data collection**	
X-ray source	NSLS-II 17-ID-1
X-ray detector	Dectris Eiger X 9M
Detector distance (mm)	131.37
Oscillation range (º)	0.2
Wavelength (Å)	0.92019
Space group	P 1 21 1
a / b / c (Å)	35.1 / 49.3 / 55.7
α / β / γ (º)	90.0 / 92.5 / 90.0
Resolution range (Å)	34.61–1.20 (1.39–1.20)
Number of observations	263271 (13826)
Unique reflections	37748 (1887)
Completeness	92.1 (51.7)
Redundancy	7.0 (7.3)
Mean $I/\sigma(I)$	10.8 (1.4)
CC1/2*	0.999 (0.412)
R_{merge}	0.07 (1.13)
R_{meas}	0.08 (1.32)
R_{pim}	0.05 (0.68)
**Refinement statistics**	
Refinement program	Phenix.refine: 1.20.1-4487-000
Resolution range (Å)	34.61–1.20
Data cutoff (sigma (F))	1.37
Completeness (working+test) (%)	63.76
Number of unique reflections refined against	37730
R-value (work)	0.1783
R-value (free)	0.1970
R-value (work + test)	0.1793
Free R value test set count	1999
Mean B value (overall $Å^2$)	22.67

Table S7. Measles Virus Fusion Glycoprotein (T461A variant): PDB 9YG6

**Data collection**	
X-ray source	NSLS-II 17-ID-1
X-ray detector	Dectris Eiger X 9M
Detector distance (mm)	190.05
Oscillation range (º)	0.1
Wavelength (Å)	0.919689
Space group	C 1 2 1
a / b / c (Å)	103.1 / 29.3 / 71.9
α / β / γ (º)	90.0 / 112.4 / 90.0
Resolution range (Å)	33.24–1.71 (1.74–1.71)
Number of observations	93403 (4765)
Unique reflections	21795 (1067)
Completeness	100.0 (100.0)
Redundancy	4.3 (4.5)
Mean $I/\sigma(I)$	7.5 (1.1)
CC1/2*	0.998 (0.377)
R_{merge}	0.08 (1.18)
R_{meas}	0.11 (1.56)
R_{pim}	0.07 (1.01)
**Refinement statistics**	
Refinement program	Phenix.refine: 1.20.1-4487-000
Resolution range (Å)	34.00–1.71
Data cutoff (sigma (F))	1.35
Completeness (working+test) (%)	45.83
Number of unique reflections refined against	18571
R-value (work)	0.2102
R-value (free)	0.2490
R-value (work + test)	0.2121
Free R value test set count	937
Mean B value (overall $Å^2$)	18.22

Table S8. Measles Virus Fusion Glycoprotein (N462K variant): PDB 9YFP

**Data collection**	
X-ray source	NSLS-II 17-ID-1
X-ray detector	Dectris Eiger X 9M
Detector distance (mm)	131.37
Oscillation range (º)	0.2
Wavelength (Å)	0.92019
Space group	P 3 2 1
a / b / c (Å)	47.7 / 47.7 / 71.4
α / β / γ (º)	90.0 / 92.5 / 120.0
Resolution range (Å)	27.02–1.86 (1.89–1.86)
Number of observations	165317 (8336)
Unique reflections	8314 (400)
Completeness	99.7 (99.3)
Redundancy	19.9 (20.8)
Mean $I/\sigma(I)$	10.3 (1.5)
CC1/2*	1.000 (0.423)
R_{merge}	0.21 (2.66)
R_{meas}	0.22 (2.79)
R_{pim}	0.07 (0.81)
**Refinement statistics**	
Refinement program	Phenix.refine: 1.20.1-4487-000
Resolution range (Å)	27.02–1.86
Data cutoff (sigma (F))	1.36
Completeness (working+test) (%)	99.53
Number of unique reflections refined against	8299
R-value (work)	0.2583
R-value (free)	0.2883
R-value (work + test)	0.2595
Free R value test set count	367
Mean B value (overall $Å^2$)	46.62

Table S9. Measles Virus Fusion Glycoprotein (G464W variant): PDB 9Y9O

**Data collection**	
X-ray source	NSLS-II 17-ID-1
X-ray detector	Dectris Eiger X 9M
Detector distance (mm)	190.05
Oscillation range (º)	0.1
Wavelength (Å)	0.91969
Space group	P 1 21 1
a / b / c (Å)	28.9 / 52.56 / 70.59
α / β / γ (º)	90.0 / 100.5 / 90.0
Resolution range (Å)	27.02–1.86 (1.89–1.86)
Number of observations	91232 (4038)
Unique reflections	24845 (1242)
Completeness	90.6 (41.0)
Redundancy	3.7 (3.3)
Mean $I/\sigma(I)$	5.7 (1.5)
CC1/2*	0.996 (0.597)
R_{merge}	0.12 (0.71)
R_{meas}	0.16 (0.99)
R_{pim}	0.11 (0.52)
**Refinement statistics**	
Refinement program	Phenix.refine: 1.20.1-4487-000
Resolution range (Å)	27.02–1.86
Data cutoff (sigma (F))	1.35
Completeness (working+test) (%)	65.78
Number of unique reflections refined against	24824
R-value (work)	0.1708
R-value (free)	0.2087
R-value (work + test)	0.1727
Free R value test set count	1270
Mean B value (overall Å^2)	19.93

Table S10. Measles Virus Fusion Glycoprotein (T461I/N462S/N465S variant): PDB 9YAC

**Data collection**	
X-ray source	NSLS-II 17-ID-1
X-ray detector	Dectris Eiger X 9M
Detector distance (mm)	131.41
Oscillation range (º)	0.2
Wavelength (Å)	0.92019
Space group	P 3
a / b / c (Å)	50.3 / 50.26 / 71.64
α / β / γ (º)	90.0 / 90.0 / 120.0
Resolution range (Å)	27.66–1.48 (1.61–1.48)
Number of observations	280821 (13376)
Unique reflections	26224 (1311)
Completeness	91.6 (50.0)
Redundancy	10.7 (10.2)
Mean $I/\sigma(I)$	9.9 (1.4)
CC1/2*	0.999 (0.345)
R_{merge}	0.13 (1.79)
R_{meas}	0.14 (2.00)
R_{pim}	0.06 (0.88)
**Refinement statistics**	
Refinement program	Phenix.refine: 1.20.1-4487-000
Resolution range (Å)	27.66–1.48
Data cutoff (sigma (F))	1.97
Completeness (working+test) (%)	99.90
Number of unique reflections refined against	25247
R-value (work)	0.1769
R-value (free)	0.2076
R-value (work + test)	0.1794
Free R value test set count	1996
Mean B value (overall $Å^2$)	29.45

Table S11. Measles Virus Fusion Glycoprotein (L454W variant): PDB 9ZUH

**Data collection**	
X-ray source	NSLS-II 17-ID-1
X-ray detector	Dectris Eiger X 9M
Detector distance (mm)	220.03
Oscillation range (º)	0.2
Wavelength (Å)	0.97934
Space group	P 1 21 1
a / b / c (Å)	57.69 / 52.09 / 72.72
α / β / γ (º)	90.0 / 91.59 / 90.0
Resolution range (Å)	57.67–2.13 (2.17–2.13)
Number of observations	65333 (3288)
Unique reflections	23573 (1152)
Completeness	96.6 (96.8)
Redundancy	2.8 (2.9)
Mean $I/\sigma(I)$	2.4 (1.2)
CC1/2*	0.967 (0.410)
R_{merge}	0.21 (0.78)
R_{meas}	0.30 (1.11)
R_{pim}	0.21 (0.78)
**Refinement statistics**	
Refinement program	Phenix.refine: 1.20.1-4487-000
Resolution range (Å)	57.67–2.13
Data cutoff (sigma (F))	1.34
Completeness (working+test) (%)	94.39
Number of unique reflections refined against	23026
R-value (work)	0.2146
R-value (free)	0.2635
R-value (work + test)	0.2188
Free R value test set count	2013
Mean B value (overall Å^2)	22.88

Classification

Table S12. Measles Virus Fusion Glycoprotein Variants Underlying Hyperfusogenicity and Structural Modulation.

MeV Variant	Hyperfusogenic/Neuropathogenic or Mechanistic Probe?	Description
Wild-type	-	Native MeV fusion protein.
L454W	Hyperfusogenic/Neuropathogenic	Identified in naturally occurring hyperfusogenic strains. [6–8]
L454M	Hyperfusogenic/Neuropathogenic	Laboratory-derived variant associated with enhanced neurovirulence and brain spread in SSPE models. [9–11]
L454A	Mechanistic Probe	Alanine substitution variant designed to probe residue-specific structural contributions.
E455G	Hyperfusogenic/Neuropathogenic	Identified in naturally occurring hyperfusogenic strains. [12]
L454W/E455G	Hyperfusogenic/Neuropathogenic	Laboratory-derived variant emerged under selective pressure. [13]
L454A/E455G	Mechanistic Probe	Alanine substitution variant incorporating combined mutation to assess cooperative effects.
T461I	Hyperfusogenic/Neuropathogenic	Identified in naturally occurring hyperfusogenic strains. [13]
T461A	Hyperfusogenic/Neuropathogenic	Laboratory-derived variant identified from hyperfusogenic mutant libraries. [14]
N462K	Hyperfusogenic/Neuropathogenic	Identified in naturally occurring hyperfusogenic strains. [14,15]
N462A	Mechanistic Probe	Alanine substitution variant designed to probe residue-specific structural contributions.
N462S	Hyperfusogenic/Neuropathogenic	Identified in naturally occurring hyperfusogenic strains. [11,16,17]
N465S	Hyperfusogenic/Neuropathogenic	Identified in naturally occurring hyperfusogenic strains. [11,17,18]
T461I/N462S	Mechanistic Probe	Rationally designed variant to evaluate cooperative contributions to fusion activity.
T461I/N465S	Mechanistic Probe	Rationally designed variant to evaluate cooperative contributions to fusion activity.
T461I/N462S/N465S	Hyperfusogenic/Neuropathogenic	Identified in naturally occurring hyperfusogenic strains. [18–20]
G464W	Hyperfusogenic/Neuropathogenic	Identified in naturally occurring hyperfusogenic strains. [15,21–23]
G464A	Mechanistic Probe	Alanine substitution variant designed to probe residue-specific structural contributions.

References

1. Kabsch, W. Integration, scaling, space-group assignment and post-refinement. *Acta Crystallogr. D Biol. Crystallogr.* 2010, *66*, 125-132.
2. Vonrhein, C., Flensburg, C., Keller, P., Sharff, A., Smart, O., Paciorek, W., Womack, T. & Bricogne, G. Data processing and analysis with the autoPROC toolbox. *Acta Cryst.* 2011, *D67*, 293-302.
3. Adams, P. D.; Afonine, P. V.; Bunkóczi, G.; Chen, V. B.; Davis, I. W.; Echols, N.; Headd, J. J.; Hung, L.-W.; Kapral, G. J.; Grosse-Kunstleve, R. W.; McCoy, A. J.; Moriarty, N. W.; Oeffner, R.; Read, R. J.; Richardson, D. C.; Richardson, J. S.; Terwilliger, T. C.; Zwart, P. H. PHENIX: a Comprehensive Python-Based System for Macromolecular Structure Solution. *Acta Crystallogr. D Biol. Crystallogr.* 2010, *66* (2), 213–221.
4. Emsley, P.; Cowtan, K. Coot: Model-Building Tools for Molecular Graphics. *Acta Crystallogr. D Biol. Crystallogr.* 2004, *60* (Pt 12 Pt 1), 2126–2132.
5. Mathieu, C.; Ferren, M.; Jurgens, E. M.; Dumont, C.; Ksenia Rybkina; Harder, O.; Stelitano, D.; Madeddu, S.; Sanna, G.; Schwartz, D.; Biswas, S.; Hardie, D.; Takao Hanabusa; Moscona, A.; Horvat, B.; Niewiesk, S.; Matteo Porotto. Measles Virus Bearing Measles Inclusion Body Encephalitis-Derived Fusion Protein Is Pathogenic after Infection via the Respiratory Route. *J. Virol.* **2019**, *93* (8).
6. Krissinel, E.; Henrick, K. Inference of macromolecular assemblies from crystalline state. *J. Mol. Biol*. **2007**, *372*, 774–797.
7. Mathieu, C.; Bovier, F. T.; Ferren, M.; Lieberman, N. A. P.; Predella, C.; Lalande, A.; Vikas Peddu; Lin, M.; Amin Addetia; Patel, A.; Outlaw, V. K.; Corneo, B.; N. Valerio Dorrello; Briese, T.; Hardie, D.; Horvat, B.; Moscona, A.; Greninger, A. L.; Matteo Porotto. Molecular Features of the Measles Virus Viral Fusion Complex That Favor Infection and Spread in the Brain. *MBio* **2021**, *12* (3).
8. Jurgens, E. M.; Mathieu, C.; Palermo, L. M.; Hardie, D.; Horvat, B.; Moscona, A.; Matteo Porotto. Measles Fusion Machinery Is Dysregulated in Neuropathogenic Variants. *MBio* **2015**, *6* (1).
9. Yousaf, I.; Domanico, L.; Toshihiko Nambara; Yadav, K.; Kelly, L. K.; Trejo-Lopez, J.; Shieh, W.-J.; Rota, P. A.; Devaux, P.; Takahisa Kanekiyo; Taylor, M. P.; Cattaneo, R. The Measles Virus Matrix F50S Mutation from a Lethal Case of Subacute Sclerosing Panencephalitis Promotes Receptor-Independent Neuronal Spread. *J. Virol.* **2024**, *99* (1).
10. Yousaf, I.; Hannon, W. W.; Donohue, R. C.; Pfaller, C. K.; Yadav, K.; Dikdan, R.; Tyagi, S.; Schroeder, D. C.; Shieh, W.; Rota, P. A.; Feder, A. F.; Cattaneo, R. Brain Tropism Acquisition: The Spatial Dynamics and Evolution of a Measles Virus Collective Infectious Unit That Drove Lethal Subacute Sclerosing Panencephalitis. *PLOS Pathog.* **2023**, *19* (12), e1011817–e1011817.
11. Watanabe, S.; Yuta Shirogane; Sato, Y.; Takao Hanabusa; Yanagi, Y. New Insights into Measles Virus Brain Infections. *Trends Microbiol.***2019**, *27* (2), 164–175.
12. Zyla, D. S.; Zipursky, G.; Marca, R. D.; Lacarbonara, D.; Niemeyer, G.; Peng, W.; Predella, C.; Stearns, K.; Leedale, C.; Miller, J.; Tay, D.; Kao, J.; Acciani, M.; Di Clemente, L.; Das, A.; Parekh, D.; Avalos, R. D.; McGill, G.; Dorrello, N. V.; Hastie, K. M.; Alabi, C. A.; Snijder, J.; Moscona, A.; Greninger, A. L.; Niewiesk, S.; Saphire, E. O.; Porotto, M. Stabilized Full-Length Measles Fusion Protein Elicits Potent Immunity and Protection In Vivo. *bioRxiv* **2025**, 2025.10.07.681039.
13. Mathieu, C.; Bovier, F. T.; Ferren, M.; Lieberman, N. A. P.; Predella, C.; Lalande, A.; Vikas Peddu; Lin, M.; Amin Addetia; Patel, A.; Outlaw, V. K.; Corneo, B.; N. Valerio Dorrello; Briese, T.; Hardie, D.; Horvat, B.; Moscona, A.; Greninger, A. L.; Porotto, M. Molecular Features of the

Measles Virus Viral Fusion Complex That Favor Infection and Spread in the Brain. *MBio* **2021**, *12* (3).

14. Hashiguchi, T.; Fukuda, Y.; Matsuoka, R.; Kuroda, D.; Kubota, M.; Shirogane, Y.; Watanabe, S.; Tsumoto, K.; Kohda, D.; Plemper, R. K.; Yanagi, Y. Structures of the Prefusion Form of Measles Virus Fusion Protein in Complex with Inhibitors. *Proc. Natl. Acad. Sci. U.S.A.* **2018**, *115* (10), 2496–2501.
15. Ikegame, S.; Hashiguchi, T.; Hung, C. T.; Dobrindt, K.; Brennand, K. J.; Takeda, M.; Lee, B. Fitness Selection of Hyperfusogenic Measles Virus F Proteins Associated with Neuropathogenic Phenotypes. *Proc. Natl. Acad. Sci. U.S.A.* **2021**, *118* (18).
16. Ha, M. N.; Sébastien Delpeut; Noyce, R. S.; Sisson, G.; Black, K. M.; Liang Tzung Lin; Bilimoria, D.; Plemper, R. K.; Privé, G. G.; Richardson, C. D. Mutations in the Fusion Protein of Measles Virus That Confer Resistance to the Membrane Fusion Inhibitors Carbobenzoxy- D -Phe- L -Phe-Gly and 4-Nitro-2-Phenylacetyl Amino-Benzamide. *J. Virol.* **2017**, *91* (23).
17. Ferren, M.; Horvat, B.; Mathieu, C. Measles Encephalitis: Towards New Therapeutics. *Viruses* **2019**, *11* (11), 1017.
18. Watanabe, S.; Yuta Shirogane; Suzuki, S.; Satoshi Ikegame; Koga, R.; Yanagi, Y. Mutant Fusion Proteins with Enhanced Fusion Activity Promote Measles Virus Spread in Human Neuronal Cells and Brains of Suckling Hamsters. *J. Virol.* **2013**, *87* (5), 2648–2659.
19. Busch, J.; Chey, S.; Sieg, M.; Vahlenkamp, T. W.; Liebert, U. G. Mutated Measles Virus Matrix and Fusion Protein Influence Viral Titer in Vitro and Neuro-Invasion in Lewis Rat Brain Slice Cultures. *Viruses* **2021**, *13* (4), 605.
20. Yuta Shirogane; Harada, H.; Hirai, Y.; Takemoto, R.; Suzuki, T.; Hashiguchi, T.; Yanagi, Y. Collective Fusion Activity Determines Neurotropism of an En Bloc Transmitted Enveloped Virus. *Sci. Adv.* **2023**, *9* (4).
21. Ikegame, S.; Hashiguchi, T.; Hung, C-T.; Dobrindt, K.; Brennand, K. J.; Takeda, M.; Lee, B. Fitness Selection of Hyperfusogenic Measles Virus F Proteins Associated with Neuropathogenic Phenotypes. *Proc. Natl. Acad. Sci. U.S.A.* **2021**, *118* (18).
22. Ayata, M.; Takeuchi, K.; Takeda, M.; Shinji Ohgimoto; Kato, S.; Luna Bhatta Sharma; Tanaka, M.; Kuwamura, M.; Ishida, H.; Ogura, H. The F Gene of the Osaka-2 Strain of Measles Virus Derived from a Case of Subacute Sclerosing Panencephalitis Is a Major Determinant of Neurovirulence. *J. Virol.* **2010**, *84* (21), 11189–11199.
23. Okada, H.; Itoh, M.; Nagata, K.; Takeuchi, K. Previously Unrecognized Amino Acid Substitutions in the Hemagglutinin and Fusion Proteins of Measles Virus Modulate Cell-Cell Fusion, Hemadsorption, Virus Growth, and Penetration Rate. *J. Virol.* **2009**, *83* (17), 8713–8721.
